# Early Pregnancy Maternal Plasma Phospholipid Saturated Fatty Acids and Fetal Growth: Findings from a Multi-Racial/Ethnic Birth Cohort in US

**DOI:** 10.3390/nu15153287

**Published:** 2023-07-25

**Authors:** Ling-Jun Li, Ruochen Du, Marion Ouidir, Ruijin Lu, Zhen Chen, Natalie L. Weir, Michael Y. Tsai, Paul S. Albert, Cuilin Zhang

**Affiliations:** 1 Department of O&G, Yong Loo Lin School of Medicine, National University of Singapore, Singapore 117575, Singapore; obgllj@nus.edu.sg; 2 Global Centre for Asian Women’s Health, Yong Loo Lin School of Medicine, National University of Singapore, Singapore 117575, Singapore; 3 NUS Bia-Echo Asia Centre for Reproductive Longevity and Equality (ARCLE), Yong Loo Lin School of Medicine, National University of Singapore, Singapore 117575, Singapore; 4 Human Potential Translation Research Programme, Yong Loo Lin School of Medicine, National University of Singapore, Singapore 117575, Singapore; 5 Biostatics Unit, Yong Loo Lin School of Medicine, National University of Singapore, Singapore 117575, Singapore; ruochen.du@nus.edu.sg; 6 Institute for Advanced Biosciences, Grenoble Aples University, Site Santé, Allée des Alpes, 38700 La Tronche, France; marion.ouidir@univ-grenoble-alpes.fr; 7 Division of Biostatistics, School of Medicine, Washington University in St. Louis, St. Louis, MO 63110, USA; r.lu@wustl.edu; 8 Division of Population Health Research, Eunice Kennedy Shriver National Institute of Child Health and Human Development, National Institutes of Health, Bethesda, MD 20892, USA; chenzhe@mail.nih.gov; 9 Department of Laboratory Medicine and Pathology, University of Minnesota, Minneapolis, MN 55455, USA; weirx065@umn.edu (N.L.W.); tsaix001@umn.edu (M.Y.T.); 10 Division of Cancer Epidemiology and Genetics, National Cancer Institute, National Institutes of Health, Bethesda, MD 20892, USA; albertp@mail.nih.gov

**Keywords:** saturated fatty acids, odd-chain fatty acids, even-chain fatty acids, very-long-chain fatty acids, fetal growth, pregnancy, plasma phospholipids

## Abstract

Saturated fatty acids (SFAs) during pregnancy are associated with disrupted metabolic programming among offspring at birth and later growth. We examined plasma phospholipid SFAs in early pregnancy and fetal growth throughout pregnancy. We enrolled 321 pregnant women from the NICHD Fetal Growth Studies—Singleton Cohort at gestational weeks 8–13. Ultrasonogram schedules were randomly assigned to capture weekly fetal growth. We measured plasma phospholipid SFAs at early pregnancy using blood samples and modeled fetal growth trajectories across tertiles of SFAs with cubic splines using linear mixed models after full adjustment. We then compared pairwise weekly fetal growth biometrics referencing the lowest tertile in each SFA using the Wald test. We found that even-chain and very long even-chain SFAs were inversely associated, whereas odd-chain SFAs were positively associated with fetal weight and size. Compared with the lowest tertile, the highest tertile of pentadecanoic acid (15:0) had a greater fetal weight and size, starting from week 13 until late pregnancy (at week 39: 3429.89 vs. 3269.08 g for estimated fetal weight; 328.14 vs. 323.00 mm for head circumference). Our findings could inspire future interventions using an alternative high-fat diet rich in odd-chain SFAs for optimal fetal growth.

## 1. Introduction

A body of evidence stemming from human and animal research has shown that maternal nutrition during pregnancy is directly related to the adequate development of the fetus and metabolism [1]. Among all nutritional factors, fatty acids have been widely investigated due to their easy transfer via placental fatty acids binding and transporting proteins to the fetus [2,3]. Emerging evidence has repeatedly reported a positive influence of the maternal antenatal dietary intake of polyunsaturated fatty acids (PUFA) on offspring birth weight and subsequent child growth [4,5,6]. Recent work from our team reported the positive temporal relationship between early pregnancy omega-3 plasma phospholipid PUFAs and fetal growth trajectories throughout pregnancy and their relevance to timing [7].

However, PUFAs are only composed of a small portion of fatty acids, leaving the majority unknown to the etiology of fetal development, such as saturated fatty acids (SFAs). SFAs are hydrogenated, solid at room temperature and highly correlated with dyslipidemia [1,8]. Animal dam models have suggested that SFAs can change the biological mechanisms of the pancreas, liver and adipose tissue in pups, such as the hypertrophy of pancreatic islets [9], proinflammatory status and insulin resistance [10] and greater body weight [11,12]. Four studies on human subjects investigated the effect of SFAs (i.e., dietary, erythrocyte levels and plasma metabolomics) on birth weight, yet the findings were equivocal. Two studies reported an inverse relationship between higher maternal erythrocyte SFAs during mid-late pregnancy and a smaller weight at birth [13,14]. On the contrary, two studies showed that maternal plasma or dietary SFAs are associated with an increased sum of skinfold at birth and a lower risk of smaller-than-gestational-age (SGA) neonates at birth [15,16]. In addition, labeling SFAs as harmful to human metabolism has been disapproved by emerging evidence because it does not differentiate the protective effect of odd-chain SFAs from the overall detrimental effect of even-chain SFAs on cardiometabolic health [17].

The existing significant knowledge gap lies in a few aspects of research on SFAs with fetal development: (1) Current studies only use birth weight as a proxy for in utero growth, which is inaccurate and may not reflect the relevance of timing in fetal growth. (2) Most observational studies assessing SFAs via dietary questionnaires are subject to self-reporting information bias [18,19]. (3) Research on maternal plasma phospholipid SFA compositions (i.e., even-chain, very long even-chain, odd-chain) during early pregnancy is sparse. Therefore, in order to understand the underlying physiology of different high-fat diets and fetal growth, as well as the relevance of timing, we explored associations and their relevance to the timing between maternal plasma phospholipid SFA compositions in early pregnancy and fetal growth trajectories throughout pregnancy, by utilizing data from a US multi-racial/ethnic birth cohort.

## 2. Materials and Methods

### 2.1. Study Population and Design

This study was based on data from the *Eunice Kennedy Shriver* National Institute of Child Health and Human Development (NICHD) Fetal Growth Studies—Singletons cohort. The prospective cohort comprised 2802 pregnant women from 12 clinical centers across the United States. Pregnant subjects were recruited if they were 18–40 years of age, were within 8–13 weeks of gestation and did not have major pre-existing chronic diseases, such as diabetes and cancer. We registered the study in the Clinical Trial Registry (NCT00912132) and published the detailed recruitment and study protocol elsewhere [20]. We included 321 women from a nested case–control study for analysis based on the primary cohort. Among them, 107 had GDM, diagnosed according to the Carpenter and Coustan criteria following the recommendations of the American College of Obstetrics and Gynecologists (ACOG), and 214 were non-GDM controls. The ratio of women with GDM and non-GDM controls was 1:2. They were matched according to maternal age (±2 years), self-reported race/ethnicity (non-Hispanic White, non-Hispanic Black, Hispanic, Asian/Pacific Islander) and gestational age (GA) at blood collection (±2 weeks).

The institutional review boards at all participating sites approved both the primary and sub-study. All participants provided written informed consent prior to data collection. The participating clinical centers entered study documents and data, including ultrasound measurements and images, into the Clinical Trial Management System, which were then electronically transferred to the Data Coordinating Center.

### 2.2. Assessments of Plasma Phospholipid Saturated Fatty Acids (SFAs)

Upon enrollment at 8–13 weeks of gestation (visit 0), we collected blood from all participants and stored biospecimens at −80 °C until thawing prior to the assay. Phospholipid fatty acid profiles were extracted using a previously described method [21,22]. Briefly, lipids were extracted with chloroform/methanol, separated using thin-layer chromatography, and the phospholipid batch was derivatized to methyl esters. The final product was injected into a capillary Varian Cp7420 30-m column with a Hewlett Packard 5890 gas chromatograph with flame ionization detection, interphased with HP Chemstation software A.06. All SFA components were calculated in percentages (%) referencing the total weight of the plasma phospholipid FA fraction. The analytic assessments and inter-assay coefficients of variation (CVs) for all SFAs in the same cohort were published elsewhere [23]. We identified eight plasma phospholipid SFAs and 3 SFA-derived indices, including the sum of even-chain SFAs: myristic acid (14:0), palmitic acid (16:0) and stearic acid (18:0); the sum of odd-chain SFAs: pentadecanoic acid (15:0) and heptadecanoic acid (17:0); and the sum of very long even-chain SFAs: arachidic acid (20:0), behenic acid (22:0) and lignoceric acid (24:0). Table S1 shows the weight of the percentages of all SFAs among all plasma phospholipid FA fractions.

### 2.3. Fetal Growth Measurement throughout Pregnancy (10–40 Weeks of Gestational Age)

Women received an ultrasonographic examination at enrollment 8–13 weeks into gestation (visit 0) and another one at four following ultrasonography schedules via randomization, as follows: weeks 16, 24, 30, 34 and 38 (group A); weeks 18, 26, 31, 35 and 39 (group B); weeks 20, 28, 32, 36 and 40 (group C); and weeks 22, 29, 33, 37 and 41 (group D), as stated in Table S2 and Figure S1. All study visits allowed ±1 week of the targeted GA to accommodate the subjects’ availability. We captured weekly fetal growth data in a mixed longitudinal randomization scheme without exposing individual women to ultrasound every week [24].

At each ultrasonographic examination, trained sonographers performed standard operating procedures using identical equipment (Voluson E8; GE Healthcare, Boston, MA, USA) and assessed a series of fetal growth biometrics, including head circumference (HC, mm), biparietal diameter (BPD, mm), abdominal circumference (AC, mm), femur length (FL, mm) and HC/AC ratio. We then calculated the estimated fetal weight (EFW, g) using a Hadlock formula based on HC, AC and FL [25]. Furthermore, measurement errors in terms of fetal growth biometrics were minimized in our study due to high inter- and intra-grader reliability reported in our study, regardless of maternal obesity status [24].

### 2.4. Covariates

At study entry, trained research coordinators interviewed participants to collect information on maternal demographics, pregnancy history and lifestyle behaviors, as well as blood pressure measurements, anthropometric indices and infant sex from medical records. We calculated GA at delivery based on the ultrasound-verified last menstrual period (LMP) of mothers and the date of delivery. Among all covariates of interest, we identified maternal pre-pregnancy body mass index (BMI) as a key covariate [26]. Despite having matched maternal age and race/ethnicity between cases and controls, we continued to control for maternal age and race/ethnicity to obtain conservative estimates. We applied a final adjustment model for all statistical analyses, including maternal age, race/ethnicity, nulliparity, pre-pregnancy BMI and infant sex.

### 2.5. Statistical Analysis

Because our study participants diagnosed with GDM (107 out of 321, 33.3%) were overrepresented compared with the general pregnant population in the US (107 out of 2802, 4%), we re-weighted all assessments following the idea of pseudolikelihood by Samuelsen (1997) [27] to represent the full cohort [24,28]. We used descriptive statistics to summarize crude and weighted characteristics of women and their neonates in the primary and nested case–control cohort (Table S3).

Tertiles of individual SFAs were treated as independent variables, and trajectories of all fetal biometrics were treated as dependent variables and modeled using a cubic spline model estimated using a restricted maximum likelihood approach [29]. Because of the skewed distribution of all fetal growth biometrics, we then log-transformed all assessments to stabilize variances across GA with an approximate normal distribution. The model initially included fixed effects of the linear, quadratic and cubic terms and cubic spline terms of GA (3 knots at the 25th, 50th and 75th percentiles), as well as a random intercept and random effects of the linear, quadratic, and cubic terms and cubic spline terms of weekly GA. The random effect covariance was unstructured, and the random effect of the cubic spline term of weekly GA was removed to facilitate model convergence.

We first applied global testing to investigate the overall difference in fetal growth trajectories across tertiles of SFAs in the full model. Then, we calculated the log–likelihood ratio by adding an interaction term between SFA tertiles and GA followed by Bonferroni correction [30]. Once a significant association was identified from all the steps mentioned above, we calculated the weekly means (back-transform fetal biometrics) and compared weekly differences in fetal growth biometrics across tertiles of each SFA (using the lowest tertile as a reference) in the full model using the Wald test. We further adjusted for family history of diabetes, maternal random glucose level at enrollment, maternal total cholesterol level at enrollment and sum of other SFA subgroups at visit 0 in the sensitivity analyses. We conducted all the analyses using SAS version 9.4 (SAS Institute, Cary, NC, USA) and R Software (version 4.2.1). We reported all estimates with a 95% confidence interval (CI) or *p*-value. We defined significance as a two-tailed *p*-value of 0.05.

## 3. Results

All SFAs and sums of SFA subgroups were significantly associated with fetal weight and size, with at least two or more fetal growth parameters, even after Bonferroni correction (Table 1 and Table S4). Among all SFAs, myristic acid (14:0), pentadecanoic acid (15:0), stearic acid (18:0), lignoceric acid (24:0) and the sum of odd-chain SFAs were consistently and significantly associated with all fetal growth biometrics (*p* < 0.05).

### 3.1. Even-Chain SFAs and Fetal Growth

Overall, plasma phospholipid even-chain SFA levels were inversely associated with fetal growth throughout pregnancy. Among three even-chain SFAs, myristic acid (14:0) and stearic acid (18:0) were associated with all fetal growth parameters, whereas palmitic acid (16:0) was only associated with AC. Compared with the lowest tertile, the highest tertile of plasma phospholipid myristic acid (14:0) was significantly associated with a decrement in FL and HC, starting in early pregnancy (FL at week 11: 4.11 vs. 4.43 mm, *p* = 0.05; HC at week 10: 47.05 vs. 55.24 mm, *p* < 0.00001) and attenuating in early-mid pregnancy (FL at week 15: 12.45 vs. 13.10 mm, *p* = 0.06; HC at week 17: 137.85 vs. 135.86 mm, *p* = 0.06) (Table S5A,B and Figure S2). Interestingly, the second tertile of myristic acid (14:0) had a stronger and longer impact on reduced EFW and AC than that of the highest tertile compared with the lowest tertile, starting in early-mid pregnancy (EFW at week 17: 174.76 vs. 181.71 g, *p* = 0.04; AC at week 13: 66.69 vs. 68.16 mm, *p* = 0.04) and attenuating in late pregnancy (EFW at week 39: 3331.94 vs. 3490.46 g, *p* = 0.08; AC at week 39: 348.05 vs. 356.81 mm, *p* = 0.09) (Table S5A and Figure S2).

Similarly, stearic acid (18:0) showed a comparable magnitude in the second tertile and highest tertiles in terms of EFW, AC, FL, HC and BPD. For example, compared with the lowest tertile, the highest tertile of stearic acid (18:0) was associated with a decrement in HC and BPD, starting from early pregnancy (HC at week 13: 83.4 vs. 85.46 mm, *p* = 0.0008; BPD at week 12: 18.86 vs. 19.35 mm, *p* = 0.04) and attenuating in mid-pregnancy (HC at week 18: 147.64 vs. 149.62 mm, *p* = 0.09; BPD at week 20: 46.37 vs. 47.06 mm, *p* = 0.10) (Table 2 and Figure S2). Compared with the lowest tertile, the second tertile of stearic acid (18:0) was also associated with a decrement yet with a longer duration in EFW and AC than that of the highest tertile, starting from early pregnancy (EFW at week 30: 1453.76 vs. 1511.69 g, *p* = 0.03; AC at week 13: 67.74 vs. 70.16 mm, *p* = 0.0005) and ending in late pregnancy (EFW: 3222.27 vs. 3731.17 g, *p* = 0.005; AC at week 40: 344.71 vs. 365.62 mm, *p* = 0.05) (Table 2 and Figure S2).

Unlike myristic acid (14:0) and stearic acid (18:0), palmitic acid (16:0) was associated with a reduced AC in the fetus during mid-pregnancy. Compared with the lowest tertile, the highest tertile of palmitic acid (16:0) was associated with a reduced AC from week 18 (125.8 vs. 129.4 mm, *p* = 0.02) to week 23 (182.41 vs. 186.54 mm, *p* = 0.07) (Table S6).

Regarding the sum of even-chain SFAs, compared with the lowest tertile, the highest tertile was significantly associated with a reduced fetal weight and size, starting from early pregnancy (EFW at week 13: 68.47 vs. 71.53 g, *p* = 0.03; AC at week 16: 102.35 vs. 105.64 mm, *p* = 0.03; FL at week 14: 9.12 vs. 9.63 mm, *p* = 0.009) to mid-pregnancy (EFW at week 19: 268.51 vs. 279.3 g, *p* = 0.07; AC at week 21: 160.32 vs. 164.22 mm, *p* = 0.08; FL at week 19: 28.33 vs. 29.15 mm, *p* = 0.15) (Table S7 and Figure S3).

### 3.2. Odd-Chain SFAs and Fetal Growth

**Figure 1:**
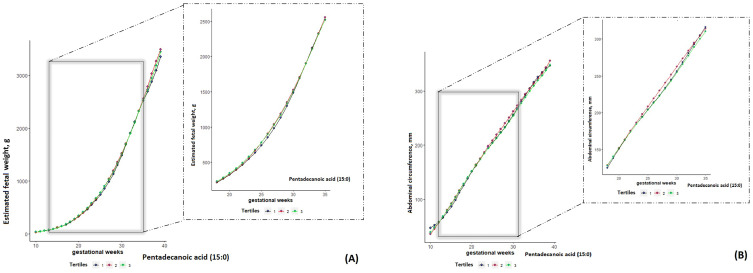
Back-transformed geometric means of estimated fetal weight and abdominal circumference by gestational weeks to tertiles of pentadecanoic acid (15:0) within the NICHD Fetal Growth Studies—Singletons cohort, 10–40 weeks of gestational age. The 1st (lowest) tertile curve is in blue, the 2nd (middle) tertile curve is in red, and the 3rd (highest) tertile is in green. The gray shaded area and the blown-up graph on the right indicate a significant increment in estimated fetal weight from 13 to 35 week of gestation (**A**), and a significant increment in abdominal circumference from 12 to 31 weeks of gestation (**B**) in both the 2nd and 3rd tertiles, compared with the 1st tertile.

In contrast to even-chain SFAs, higher levels of individual and subgroup odd-chain SFAs were associated with a larger fetal weight and size. Compared with the lowest tertile, the highest tertile of pentadecanoic acid (15:0) was associated with a larger EFW, AC, FL, HC and BPD throughout pregnancy, starting from early pregnancy (EFW at week 13: 73.22 vs. 67.03 g, *p* < 0.00001; AC at week 12: 59.13 vs. 56.77 mm, *p* = 0.002; FL at week 16.16 vs. 15.49 mm, *p* = 0.05; HC at week 13: 87.23 vs. 83.29 mm, *p* < 0.00001; BPD at week 12: 19.97 vs. 19.44 mm, *p* = 0.02) and attenuating in late pregnancy (EFW at week 36: 2778 vs. 2703.66 g, *p* = 0.14; AC at week 32: 282.88 vs. 277.97 mm, *p* = 0.07; FL at week 19: 29.12 vs. 28.06, *p* = 0.07; HC at week 39: 328.14 vs. 323 mm, *p* = 0.13; BPD at week 40: 92.66 vs. 88.91 mm, *p* = 0.07) (Table 3 and Figure 1).

**Figure 2:**
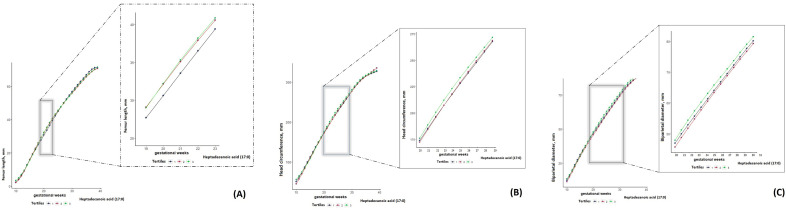
Back-transformed geometric means of femur length, head circumference and biparietal diameter by gestational weeks to tertiles of heptadecanoic acid (17:0) within the NICHD Fetal Growth Studies—Singletons cohort, 10–40 weeks gestational age. The 1st (lowest) tertile curve is in blue, the 2nd (middle) tertile curve is in red, and the 3rd (highest) tertile is in green. The gray shaded area and the blown-up graph on the right indicate a significant increment in estimated femur length from 19 to 23 week of gestation (**A**), a significant increment in head circumference from 20 to 29 weeks of gestation (**B**), and a significant increment in biparietal diameter from 20 to 32 weeks of gestation (**C**) in both the 2nd and 3rd tertiles, compared with the 1st tertile.

Even though there were similar effect sizes, heptadecanoic acid (17:0) exerted a relatively smaller impact on fetal growth than that of pentadecanoic acid (15:0). For instance, compared with the lowest tertile, the highest tertile of heptadecanoic acid (17:0) was associated with EFW, AC, FL, HC and BPD since early-mid pregnancy, yet it attenuated in mid-late pregnancy (EFW at week 23: 580.65 vs. 555.66 g, *p* = 0.10; AC at week 23: 188.71 vs. 184.96 mm, *p* = 0.12; FL at week 24: 43.31 vs. 42.02 mm, *p* = 0.09; HC at week 30: 281.54 vs. 276.73 mm, *p* = 0.06; BPD at week 33: 83.69 vs. 82.08 mm, *p* = 0.07) (Table S8 and Figure 2).

By combing pentadecanoic acid (15:0) and heptadecanoic acid (17:0), the sum of odd-chain SFAs was found to be significant with a larger EFW, FL, HC and BPD, but not with AC. Compared with the lowest tertile, the highest tertile of the sum of SFAs was associated with a greater fetal weight and size, only being significant starting from mid-pregnancy (EFW at week 17: 185.81 vs. 178.43 g, *p* = 0.03; FL at week 17: 23.14 vs. 22.03 mm, *p* = 0.02; HC at week 21: 187.27 vs. 184.11 mm, *p* = 0.03; BPD at week 18: 37.49 vs. 36.9 mm, *p* = 0.02) until the end of pregnancy (Table S9).

### 3.3. Very Long Even-Chain SFAs and Fetal Growth

Overall, higher levels of arachidic acid (20:0), behenic acid (22:0) and lignoceric acid (24:0) were associated with a reduced fetal weight and/or size. Compared with the lowest tertile, the highest tertile of lignoceric acid (24:0) was significantly associated with a reduced AC and BPD since early-mid pregnancy (AC at week 29: 257.99 vs. 265.45 mm, *p* = 0.01; BPD at week 18: 40.07 vs. 40.89 mm, *p* = 0.04), and such associations attenuated at the end of pregnancy (AC at week 40: 357.71 vs. 374.16 mm, *p* = 0.07; BPD at week 40: 87.08 vs. 93.7 mm, *p* = 0.001) (Table S10 and Figure S4A). Interestingly, the impacts of arachidic acid (20:0) and behenic acid (22:0) on reduced fetal weight and size were much weaker and shorter in duration (Tables S11 and S12).

In terms of the sum of very long even-chain SFAs, the impact on reduced fetal weight and size was more significant and longer in duration in the second tertile than that in the highest tertile, compared with the lowest tertile. For example, women in the second tertile of the sum of very long even-chain SFAs exerted an early-pregnancy decrement in fetal growth parameters (EFW at week 13: 68.5 vs. 71.38 g, *p* = 0.007; AC at week 10: 42.46 vs. 38.31 mm, *p* = 0.03; HC at week 13: 83.94 vs. 85.82 mm, *p* = 0.005; BPD at week 12: 19.22 vs. 19.68 mm, *p* = 0.03) in reference to the lowest tertile. All significant associations lasted until late pregnancy (EFW at week 30: 1463.47 vs. 1529.36 g, *p* = 0.08; AC at week 33: 290.07 vs. 295.33 mm, *p* = 0.06; HC at week 36: 315.98 vs. 320.83 mm, *p* = 0.08; BPD at week 34: 83.39 vs. 84.85 mm, m = 0.07) (Table S13 and Figure S4B).

The sensitivity analysis of additional adjustments on family history of diabetes, maternal plasma random glucose levels, total cholesterol levels and the sum of other subgroups of SFAs at visit 0 in the global test did not attenuate any significant associations in the relationships identified above (Tables S14–S17).

## 4. Discussion

Our prospective longitudinal data suggest opposite associations of subgroups of SFAs with fetal growth throughout pregnancy. Specifically, higher maternal plasma phospholipid odd-chain SFAs in early pregnancy were positively associated with fetal growth. In contrast, higher maternal plasma phospholipid even-chain and very long even-chain SFAs were inversely associated with fetal growth in early pregnancy. The relevance of timing in fetal growth increments or decrements with different subgroups of SFAs significantly started in early-mid pregnancy (10–15 weeks of gestation) and attenuated in mid-late pregnancy (23–40 weeks of gestation).

Traditional evidence indicates that SFAs are strongly associated with impaired insulin sensitivity, glucose intolerance and lipotoxicity, which could be biased by failing to separate the metabolic beneficial components, such as odd-chain SFAs, from conventional cardiometabolic risk components, such as even-chain SFAs [17]. However, instead of avoiding all high-fat diets, emerging research investigated the fat composition and differentiated the good high-fat pattern from the bad [31]. For example, maternal *n*-3 polyunsaturated fatty acids (PUFAs) levels were proven to be beneficial not only for cardiometabolic health in the general population [32] but also for fetal growth throughout pregnancy [33]. However, current evidence only focuses on growth biometrics after delivery, and the assessment of maternal SFAs is diverse in techniques and gestational age. Therefore, studies regarding plasma phospholipid maternal SFA compositions and fetal growth are sparse in the research scope.

Even-chain SFAs are FAs that are totally hydrogenated, with a linear chain without double bonds between carbon atoms. They are in a stable state at room temperature and include lauric acid (12:0), myristic acid (14:0), palmitic acid (16:0) and stearic acid (18:0) [1]. All even-chain SFAs can be derived from both exogenous intake (e.g., Western diets rich in butter, palm oil and red meat) and endogeneous synthesis (e.g., the de novo lipogenesis [DNL] pathway) [34,35], from which palmitic acid (16:0) and stearic acid (18:0) were mainly synthesized. Such even-chain FAs seemed to mediate multiple biological mechanisms, including increasing oxidative stress [17], inducing insulin resistance via the proteasomal degradation of key insulin-signaling molecules [36] and activating proinflammatory signaling via Toll-like receptor 4 [37]. Our study found that the total sum of maternal even-chain SFAs during early pregnancy was associated with reduced fetal growth from early to mid-late pregnancy. Among them, myristic acid (14:0) and stearic acid (18:0) imposed a much stronger impact on fetal weight and size than that of palmitic acid (16:0). We speculated two possible underlying pathophysiological mechanisms to our observation. First, increased levels of different even-chain SFAs might be attributable to elevated levels of oxidative stress and inflammation in vivo [17,38], and such changes could viciously disrupt the placental circulation and supply of nutrients and oxygen that are vital for fetal growth. Second, it is suggested that placental tissue has lipoprotein receptors and expresses enzymes with lipase and phospholipase activities [39,40,41]. Such activities were involved in the mechanism of maternal FA transfer across the placenta to sustain the fetal lipid requirements. In other words, the elevation of even-chain SFAs might compete with other beneficial yet much larger molecules of FAs (e.g., *n*-3 PUFAs) through the placenta, indirectly contributing to inadequate fetal growth [13,14].

The origin of odd-chain SFAs has long been attributed to the diet, especially dairy product intake [42]. However, emerging evidence suggests that circulating pentadecanoic acid (15:0) and heptadecanoic acid (17:0) are independently derived. For instance, pentadecanoic acid (15:0) correlated directly with dietary intake, and heptadecanoic acid (17:0) is a product of biosynthesis regulated by dietary intake [43,44]. Several epidemiological studies have shown that both pentadecanoic acid (15:0) and heptadecanoic acid (17:0) have protective effects on glucose homeostasis via the inhibition of hepatic oxidation [45]. Therefore, odd-chain SFAs are inversely associated with metabolic diseases [46,47,48], including reducing the risk of type 2 diabetes (T2D) [17]. Our findings indicate a beneficial impact of elevated maternal plasma phospholipid odd-chain SFAs during early pregnancy and fetal growth throughout pregnancy, indirectly aligned with the optimal effect of odd-chain SFAs suggested a priori. For example, Santaren et al. suggested that circulating pentadecanoic acid (15:0) is inversely associated with plasminogen activator inhibitor-1 (PAI-1), tumor necrosis factor-α (TNF-α) and interleukin-18 (IL-18) [49]. In another study by Zheng et al., research findings also indicated inverse associations between higher levels of odd-chain SFAs and lower levels of major lipids (i.e., total cholesterol, triglycerides, apolipoprotein A-1 and apolipoprotein B) and hepatic markers [50]. We postulate that odd-chain SFAs reduced the inflammation levels both in the maternal environment and placental perfusion, contributing to sufficient fetal nutrients and blood supply.

Regarding very long even-chain SFAs, they have not been extensively studied except for their known roles in hereditary peroxisomal disorders and special food formulations [51]. Common knowledge on very long even-chain SFAs suggests that they are derived from a limited food source, such as peanuts, macadamia nuts and canola oil [52], and from endogenous metabolism from stearic acid (18:0) [53]. Studies have shown that very long even-chain SFAs are the major components of insulin resistance and reduced β-cell mass and function [52,54,55]. Our cohort shows that the sum of very long even-chain SFAs, especially lignoceric acid (24:0), is associated with a reduced fetal weight and size, which might be related to inflammation and disrupted glycemic metabolism. However, emerging evidence suggests that circulating very long even-chain SFAs are protective against cardiovascular outcomes, such as incident heart failure, atrial fibrillation, coronary heart disease, sudden cardiac arrest and even better aging [51]. Further research on such SFA clusters regarding their biological functions and impacts on fetal growth and child health is warranted.

### 4.1. Clinical Implications

Our study investigated the relationship between maternal plasma phospholipid SFAs in early pregnancy and fetal growth trajectories throughout pregnancy and further identified the relevance of timing for significant associations, in a relatively healthier pregnant population in the US with a lower prevalence of GDM (~4%). Such results remain significant even after adjustment for family history of diabetes, maternal early pregnancy glucose, total cholesterol levels and other subgroups of SFAs. Therefore, findings from the present study are robust even for a generally healthy population with fewer pregnancy complications. Further, this study is able to differentiate the physiological roles of even-chain, odd-chain and very long even-chain SFAs underlying fetal growth throughout pregnancy. Most of our observations on fetal growth were significant from early to mid-pregnancy, a stage of which provided useful information for the development of pre-eclampsia and pre-term birth [56] and even for the assessment of weight at birth [57]. Key findings regarding the beneficial impact of pentadecanoic acid (15:0) and heptadecanoic acid (17:0) in fetal growth can inspire future directions in oral supplementation. Thus, our data might offer evidence to support the targeting of plasma phospholipid odd-chain SFAs in early pregnancy to benefit fetal growth in the general population with pregnancy.

### 4.2. Strengths and Limitations

Our study has a few notable strengths. The prospective and longitudinal data collection included plasma phospholipid SFAs, abundant forms of circulating SFAs representing both exogenous and endogenous sources of SFAs, and longitudinal fetal growth ultrasound measures. We reported the temporal relationship between plasma phospholipid SFAs in early pregnancy and fetal growth throughout gestation and also identified the relevance of timing for significant associations. In addition, such a comprehensive panel of plasma phospholipid SFAs may further help elucidate the different roles of SFAs underlying the physiology of fetal development.

However, our study was not without limitations. Even though this is one of the largest prospective pregnancy cohorts investigating fetal growth throughout pregnancy, the relatively small sample size of 321 subjects may limit the statistical power of identifying the relevance of timing using pairwise comparisons at each gestation week. Second, we cannot eliminate residual confounding due to the study’s observational nature, even though we controlled for known major confounders in our modeling. Third, dietary patterns or other lifestyle factors in mid-to-late pregnancy might modify the associations found in our study. However, such factors collected via dietary questionnaires are prone to measurement errors due to the subjectivity of self-reporting, and adjusting such factors could incur collider bias because they could be the potential mediators for subsequent fetal growth. Fourth, our observations were found to be significant mostly during early to mid-pregnancy instead of late pregnancy. It could be affected by the reduced power of fetal growth biometrics comparisons between tertiles because subjects gave birth at term. Last, there could be interactive effects among SFAs or between SFAs and other types of FAs, even though we accounted for the confounding effects among subgroups of SFAs for individual SFAs investigated in this study. Further studies with a larger sample size should investigate the underlying interaction among all FAs and are also warranted to verify our findings.

## 5. Conclusions

In summary, our study differentiates the inverse associations of even-chain and very long even-chain SFAs, in contrast to the positive associations of odd-chain SFAs in early pregnancy with fetal growth throughout pregnancy. Considering the modifiable nature of plasma phospholipid odd-chain SFAs due to their exogenous origins, such as pentadecanoic acid (15:0), our data might provide a potential target toward odd-chain SFAs (e.g., via oral supplements) to benefit fetal growth during pregnancy.

## Figures and Tables

**Table 1:** Summary of significant (Bonferroni corrected *p*-values < 0.05) impact of increased plasma phospholipid saturated fatty acids during early pregnancy on individual fetal biometric velocity throughout pregnancy (10–40 weeks of gestation).

Saturated Fatty Acids	EFW	AC	FL	HC	BPD
Myristic acid (14:0)	**↓**	**↓**	**↓**	**↓**	**↓**
Pentadecanoic acid (15:0)	**↑**	**↑**	**↑**	**↑**	**↑**
Palmitic acid (16:0)	n.s.	↓	n.s.	n.s.	n.s.
Heptadecanoic acid (17:0)	**↑**	**↑**	**↑**	**↑**	**↑**
Stearic acid (18:0)	**↓**	**↓**	**↓**	**↓**	**↓**
Arachidic acid (20:0)	n.s.	n.s.	**↓**	**↓**	**↓**
Behenic acid (22:0)	↓	↓	n.s.	↓	↓
Lignoceric acid (24:0)	↓	↓	↓	↓	↓
Sum of even-chain SFAs (14:0 + 16:0 + 18:0)	↓	↓	↓	↓	↓
Sum of odd-chain SFAs (15:0 + 17:0)	↑	n.s.	↑	↑	↑
Sum of very long even-chain SFAs (20:0 + 22:0 + 24:0)	↓	↓	n.s.	↓	↓

Abbreviations: EFW, estimated fetal weight; AC, abdominal circumference; FL, femur length; HC, head circumference; BPD, biparietal diameter; n.s., non-significant. “↑” indicates a positive association. “↓” indicates an inverse association.

**Table 2:** Back-transformed and pairwise comparison of weekly fetal growth biometrics across stearic acid (18:0) tertiles in the NICHD Fetal Growth Studies—Singletons cohort.

GW	Estimated Fetal Weight (EFW), g					Abdominal Circumference (AC), mm					Femur Length (FL), mm				
	Back-Transformed Geometric Mean g			Wald Test for Pairwise Comparison *p*-Value		Back-Transformed Geometric Mean mm			Wald Test for Pairwise Comparison *p*-Value		Back-Transformed Geometric Mean mm			Wald Test for Pairwise Comparison *p*-Value	
	1st Tertile	2nd Tertile	3rd Tertile	2nd vs. 1st Tertile	3rd vs. 1st Tertile	1st Tertile	2nd Tertile	3rd Tertile	2nd vs. 1st Tertile	3rd vs. 1st Tertile	1st Tertile	2nd Tertile	3rd Tertile	2nd vs. 1st Tertile	3rd vs. 1st Tertile
10	35.13	36.03	40.58	0.715	0.061	36.05	39.4	41.87	0.059	0.004	2.01	2.42	2.47	0.002	<0.001
11	43.92	44.69	47.72	0.628	0.049	46.55	47.87	49.18	0.258	0.050	3.76	4.26	4.17	<0.001	0.004
12	55.41	56.03	57.51	0.552	0.071	58.04	57.34	57.49	0.328	0.482	6.26	6.78	6.47	<0.0001	0.199
13	70.34	70.77	70.67	0.724	0.749	70.16	67.74	66.81	<0.001	<0.0001	9.41	9.84	9.31	0.017	0.661
14	89.54	89.74	88.09	0.913	0.348	82.55	78.95	77.12	0.0001	<0.0001	12.93	13.20	12.55	0.313	0.199
15	113.93	113.85	110.79	0.970	0.142	94.88	90.82	88.35	<0.001	<0.0001	16.54	16.61	15.99	0.819	0.158
16	144.43	144.03	139.87	0.880	0.082	106.94	103.17	100.36	<0.001	<0.0001	19.97	19.87	19.45	0.801	0.260
17	181.81	181.07	176.34	0.815	0.074	118.65	115.76	112.95	0.016	<0.0001	23.12	22.89	22.8	0.591	0.539
18	226.55	225.46	220.87	0.788	0.121	130.09	128.38	125.84	0.203	<0.001	26.06	25.75	26.00	0.509	0.918
19	278.72	277.29	273.47	0.783	0.249	141.4	140.77	138.69	0.676	0.049	28.99	28.60	29.12	0.472	0.825
20	338.68	336.91	334.13	0.780	0.415	152.59	152.79	151.26	0.901	0.369	31.91	31.46	32.19	0.448	0.677
21	406.89	404.73	403.28	0.771	0.587	163.59	164.35	163.45	0.651	0.927	34.79	34.29	35.16	0.428	0.609
22	483.83	481.12	481.37	0.750	0.751	174.39	175.38	175.19	0.554	0.605	37.59	37.07	38.01	0.415	0.598
23	570.04	566.42	568.87	0.713	0.897	185.00	185.91	186.47	0.595	0.354	40.30	39.75	40.71	0.415	0.630
24	666.15	660.99	666.34	0.660	0.986	195.46	195.98	197.32	0.775	0.267	42.90	42.33	43.25	0.433	0.688
25	772.94	765.21	774.49	0.586	0.905	205.83	205.68	207.83	0.942	0.275	45.36	44.79	45.66	0.462	0.756
26	891.44	879.55	894.24	0.485	0.857	216.21	215.17	218.11	0.634	0.343	47.70	47.13	47.94	0.491	0.814
27	1022.98	1004.62	1026.83	0.354	0.833	226.74	224.64	228.36	0.362	0.448	49.92	49.33	50.13	0.505	0.841
28	1169.33	1141.22	1173.91	0.203	0.826	237.57	234.31	238.79	0.153	0.574	52.02	51.41	52.28	0.496	0.818
29	1332.26	1290.44	1337.56	0.082	0.818	248.84	244.36	249.65	0.040	0.699	54.04	53.39	54.43	0.463	0.730
30	1511.69	1453.76	1518.15	0.032	0.802	260.44	254.82	260.96	0.010	0.797	55.98	55.28	56.61	0.439	0.600
31	1706.19	1633.15	1713.46	0.023	0.808	272.16	265.72	272.52	0.007	0.869	57.86	57.14	58.75	0.453	0.481
32	1913.17	1831.17	1919.6	0.032	0.857	283.76	277.07	284.05	0.014	0.909	59.71	59.01	60.82	0.503	0.408
33	2128.69	2051.11	2130.77	0.077	0.960	294.98	288.87	295.25	0.038	0.922	61.55	60.94	62.75	0.581	0.395
34	2347.34	2297.17	2339.22	0.292	0.858	305.51	301.17	305.78	0.130	0.921	63.42	62.99	64.49	0.704	0.465
35	2563.79	2567.88	2536.39	0.938	0.575	315.13	313.79	315.28	0.639	0.955	65.31	65.21	65.98	0.929	0.663
36	2778.72	2841.97	2724.29	0.315	0.329	324.18	325.9	323.93	0.614	0.936	67.13	67.43	67.22	0.804	0.956
37	2995.99	3086.12	2914.31	0.221	0.210	333.14	336.32	332.4	0.424	0.834	68.72	69.39	68.30	0.626	0.803
38	3221.46	3258.69	3121.99	0.633	0.163	342.6	343.79	341.43	0.761	0.746	69.93	70.81	69.33	0.528	0.732
39	3463.07	3315.86	3367.49	0.126	0.229	353.19	346.99	351.89	0.202	0.741	70.59	71.37	70.42	0.631	0.925
40	3731.17	3222.27	3677.29	0.005	0.685	365.62	344.71	364.73	0.053	0.914	70.56	70.82	71.69	0.933	0.614
**GW**	**Head Circumference (HC), mm**					**Biparietal Diameter (BPD), mm**					**HC/AC Ratio**				
	**Back-Transformed Geometric Mean** **mm**			**Wald Test for Pairwise Comparison** ***p*-Value**		**Back-Transformed Geometric Mean** **mm**			**Wald Test for Pairwise Comparison** ***p*-Value**		**Back-Transformed Geometric Mean**			**Wald Test for Pairwise Comparison** ***p*-Value**	
	**1st Tertile**	**2nd Tertile**	**3rd Tertile**	**2nd vs. 1st Tertile**	**3rd vs. 1st Tertile**	**1st Tertile**	**2nd Tertile**	**3rd Tertile**	**2nd vs. 1st Tertile**	**3rd vs. 1st Tertile**	**1st Tertile**	**2nd Tertile**	**3rd Tertile**	**2nd vs. 1st Tertile**	**3rd vs. 1st Tertile**
10	46.38	51.84	49.12	<0.001	0.141	11.36	12.86	12.24	<0.001	0.111	1.27	1.31	1.18	0.338	0.041
11	58.7	62.51	59.75	0.0001	0.402	15.21	16.13	15.42	0.003	0.580	1.26	1.31	1.22	0.066	0.080
12	71.87	73.99	71.25	<0.001	0.394	19.35	19.64	18.86	0.131	0.036	1.25	1.29	1.24	<0.001	0.427
13	85.46	86.10	83.40	0.303	<0.001	23.55	23.29	22.43	0.174	<0.0001	1.24	1.27	1.25	<0.0001	0.119
14	99.05	98.64	96.02	0.613	<0.001	27.61	26.99	26.06	0.014	<0.0001	1.22	1.25	1.24	0.004	0.011
15	112.36	111.42	108.90	0.319	<0.001	31.39	30.64	29.66	0.011	<0.0001	1.21	1.23	1.23	0.034	0.009
16	125.19	124.26	121.87	0.338	0.003	34.84	34.20	33.17	0.037	<0.0001	1.19	1.21	1.21	0.130	0.017
17	137.56	137.04	134.81	0.604	0.014	38.00	37.65	36.57	0.263	<0.0001	1.18	1.18	1.19	0.419	0.069
18	149.62	149.66	147.64	0.975	0.090	41.00	40.98	39.88	0.961	0.002	1.17	1.17	1.17	0.929	0.369
19	161.63	162.06	160.34	0.727	0.311	44.01	44.24	43.14	0.551	0.027	1.16	1.15	1.16	0.634	0.988
20	173.56	174.16	172.87	0.651	0.612	47.06	47.42	46.37	0.385	0.096	1.15	1.14	1.14	0.357	0.463
21	185.33	185.88	185.12	0.689	0.881	50.13	50.50	49.54	0.380	0.170	1.14	1.13	1.13	0.197	0.190
22	196.89	197.18	197.03	0.830	0.921	53.20	53.47	52.65	0.514	0.206	1.14	1.12	1.12	0.118	0.079
23	208.16	208.05	208.54	0.940	0.799	56.24	56.34	55.68	0.819	0.203	1.13	1.12	1.12	0.096	0.042
24	219.11	218.50	219.62	0.693	0.743	59.24	59.11	58.62	0.777	0.184	1.13	1.12	1.11	0.127	0.039
25	229.7	228.59	230.28	0.507	0.733	62.18	61.82	61.48	0.465	0.169	1.12	1.11	1.11	0.236	0.058
26	239.92	238.4	240.53	0.401	0.741	65.02	64.48	64.26	0.304	0.165	1.12	1.11	1.10	0.470	0.108
27	249.78	248.03	250.44	0.360	0.738	67.77	67.13	66.97	0.250	0.173	1.11	1.11	1.10	0.879	0.200
28	259.29	257.63	260.06	0.385	0.701	70.39	69.81	69.64	0.292	0.201	1.10	1.10	1.09	0.548	0.353
29	268.47	267.25	269.49	0.509	0.604	72.87	72.54	72.26	0.525	0.284	1.08	1.09	1.08	0.100	0.589
30	277.28	276.73	278.69	0.770	0.467	75.22	75.27	74.85	0.916	0.504	1.07	1.09	1.07	0.006	0.912
31	285.67	285.91	287.51	0.909	0.369	77.45	77.95	77.36	0.372	0.876	1.05	1.08	1.05	<0.001	0.771
32	293.58	294.56	295.77	0.668	0.334	79.58	80.52	79.74	0.141	0.810	1.04	1.06	1.04	<0.001	0.571
33	300.97	302.48	303.30	0.530	0.342	81.65	82.90	81.94	0.062	0.668	1.02	1.05	1.03	0.002	0.455
34	307.79	309.43	309.89	0.485	0.396	83.66	85.02	83.93	0.035	0.703	1.01	1.03	1.01	0.013	0.370
35	313.98	315.23	315.37	0.597	0.564	85.64	86.81	85.64	0.066	0.997	1.00	1.00	1.00	0.356	0.302
36	319.41	319.81	319.79	0.883	0.885	87.50	88.24	87.08	0.327	0.569	0.98	0.98	0.99	0.561	0.276
37	323.93	323.15	323.39	0.795	0.856	89.10	89.26	88.26	0.850	0.336	0.97	0.96	0.98	0.255	0.233
38	327.41	325.27	326.46	0.472	0.758	90.33	89.87	89.23	0.582	0.219	0.95	0.95	0.96	0.568	0.114
39	329.69	326.16	329.28	0.334	0.898	91.04	90.02	90.03	0.335	0.272	0.93	0.94	0.94	0.119	0.052
40	330.64	325.86	332.16	0.515	0.788	91.13	89.72	90.69	0.541	0.810	0.89	0.95	0.91	0.009	0.214

HC/AC ratio, head circumference/abdominal circumference ratio.

**Table 3:** Back-transformed and pairwise comparison of weekly fetal growth biometrics across pentadecanoic acid (15:0) tertiles in the NICHD Fetal Growth Studies—Singletons cohort.

GW	Estimated Fetal Weight (EFW), g					Abdominal Circumference (AC), mm					Femur Length (FL), mm				
	Back-Transformed Geometric Mean g			Wald Test for Pairwise Comparison *p*-Value		Back-Transformed Geometric Mean mm			Wald Test for Pairwise Comparison *p*-Value		Back-Transformed Geometric Mean			Wald Test for Pairwise Comparison *p*-Value	
	1st Tertile	2nd Tertile	3rd Tertile	2nd vs. 1st Tertile	3rd vs. 1st Tertile	1st Tertile	2nd Tertile	3rd Tertile	2nd vs. 1st Tertile	3rd vs. 1st Tertile	1st Tertile	2nd Tertile	3rd Tertile	2nd vs. 1st Tertile	3rd vs. 1st Tertile
10	41.85	32.27	35.96	0.001	0.109	43.76	36.67	38.62	<0.001	0.021	2.68	2.14	2.57	<0.001	0.525
11	47.07	42.06	45.36	0.008	0.461	49.66	46.25	48.40	0.005	0.370	4.37	3.9	4.33	0.002	0.782
12	55.24	54.46	57.54	0.520	0.083	56.77	56.82	59.13	0.948	0.002	6.59	6.38	6.67	0.169	0.602
13	67.03	70.05	73.22	0.021	<0.0001	65.17	68.13	70.59	<0.0001	<0.0001	9.27	9.47	9.53	0.321	0.176
14	83.38	89.46	93.22	0.001	<0.0001	74.88	79.96	82.52	<0.0001	<0.0001	12.29	12.95	12.76	0.024	0.086
15	105.35	113.42	118.45	0.001	<0.0001	85.83	92.07	94.68	<0.0001	<0.0001	15.49	16.52	16.16	0.006	0.058
16	134.02	142.69	149.83	0.003	<0.0001	97.84	104.28	106.87	<0.0001	<0.0001	18.71	19.94	19.55	0.005	0.045
17	170.13	178.10	188.18	0.025	<0.0001	110.55	116.46	118.94	<0.0001	<0.0001	21.88	23.08	22.83	0.014	0.043
18	213.64	220.47	234.09	0.125	<0.0001	123.42	128.58	130.81	<0.0001	<0.0001	24.98	25.99	25.99	0.066	0.054
19	263.96	270.61	287.73	0.235	<0.0001	135.99	140.67	142.49	0.001	<0.0001	28.06	28.83	29.12	0.212	0.072
20	321.22	329.34	349.24	0.233	<0.0001	148.1	152.67	153.95	0.004	<0.001	31.09	31.67	32.21	0.391	0.085
21	385.65	397.50	419.03	0.143	<0.0001	159.65	164.49	165.14	0.003	0.001	34.02	34.49	35.22	0.529	0.091
22	457.56	475.86	497.51	0.054	<0.0001	170.60	176.09	176.03	0.001	0.002	36.84	37.25	38.11	0.600	0.093
23	537.39	565.13	585.13	0.013	<0.0001	181.00	187.43	186.61	0.0001	0.001	39.51	39.93	40.86	0.608	0.095
24	625.81	665.91	682.42	0.003	<0.0001	190.94	198.52	196.94	<0.0001	0.001	42.04	42.53	43.45	0.575	0.101
25	723.81	778.66	790.04	<0.001	<0.0001	200.59	209.38	207.09	<0.0001	0.001	44.43	45.03	45.89	0.525	0.116
26	832.86	903.68	908.87	<0.001	<0.0001	210.16	220.08	217.15	<0.0001	0.002	46.72	47.44	48.18	0.477	0.140
27	955.00	1041.11	1040.08	<0.001	<0.0001	219.93	230.70	227.27	<0.0001	0.002	48.94	49.77	50.35	0.445	0.177
28	1093.05	1190.85	1185.24	<0.001	<0.0001	230.24	241.37	237.64	<0.0001	0.002	51.14	52.02	52.44	0.437	0.234
29	1249.44	1352.64	1346.23	<0.001	<0.0001	241.38	252.19	248.44	<0.0001	0.002	53.38	54.22	54.48	0.472	0.328
30	1424.33	1526.24	1523.6	0.001	<0.0001	253.23	263.13	259.70	<0.0001	0.003	55.65	56.36	56.5	0.553	0.471
31	1616.67	1711.36	1716.02	0.010	<0.0001	265.53	274.09	271.24	<0.001	0.016	57.92	58.46	58.47	0.667	0.656
32	1824.08	1907.67	1921.04	0.054	0.003	277.97	284.97	282.88	0.008	0.072	60.13	60.52	60.39	0.778	0.845
33	2042.57	2114.8	2134.87	0.149	0.014	290.17	295.64	294.36	0.060	0.162	62.25	62.54	62.26	0.847	0.997
34	2266.30	2332.40	2352.27	0.231	0.030	301.70	305.99	305.42	0.138	0.214	64.23	64.52	64.07	0.847	0.909
35	2488.31	2559.63	2567.07	0.234	0.055	312.14	315.92	315.78	0.170	0.208	66.01	66.48	65.81	0.760	0.888
36	2703.66	2791.87	2778.00	0.197	0.137247	321.54	325.70	325.45	0.185	0.238	67.58	68.4	67.48	0.619	0.947
37	2908.15	3021.68	2988.34	0.152	0.194177	330.17	335.75	334.58	0.139	0.259	68.93	70.23	69.09	0.466	0.925
38	3097.81	3239.88	3203.25	0.102	0.104549	338.36	346.56	343.40	0.036	0.201	70.05	71.94	70.64	0.306	0.733
39	3269.08	3435.79	3429.89	0.076	0.039051	346.46	358.67	352.13	0.003	0.211	70.94	73.48	72.15	0.194	0.522
40	3418.87	3597.76	3677.57	0.171	0.129976	354.87	372.73	361.06	0.026	0.524	71.61	74.8	73.62	0.272	0.483
**GW**	**Head Circumference (HC), mm**					**Biparietal Diameter (BPD), mm**					**HC/AC Ratio**				
	**Back-Transformed Geometric Mean** **mm**			**Wald Test for Pairwise Comparison** ***p*-Value**		**Back-Transformed Geometric Mean mm**			**Wald Test for Pairwise Comparison** ***p*-Value**		**Back-Transformed Geometric Mean**			**Wald Test for Pairwise Comparison ** ***p*-Value**	
	**1st Tertile**	**2nd Tertile**	**3rd Tertile**	**2nd vs. 1st Tertile**	**3rd vs. 1st Tertile**	**1st Tertile**	**2nd Tertile**	**3rd Tertile**	**2nd vs. 1st Tertile**	**3rd vs. 1st Tertile**	**1st Tertile**	**2nd Tertile**	**3rd Tertile**	**2nd vs. 1st Tertile**	**3rd vs. 1st Tertile**
10	58.71	43.93	52.76	<0.0001	0.013	14.20	10.88	13.10	<0.0001	0.096	1.33	1.18	1.39	<0.001	0.348
11	65.57	56.76	63.50	<0.0001	0.155	16.69	14.64	16.42	<0.0001	0.522	1.32	1.22	1.32	<0.0001	0.870
12	73.76	70.50	75.05	0.0001	0.096	19.44	18.73	19.97	0.001	0.021	1.30	1.24	1.27	<0.0001	0.053
13	83.29	84.60	87.23	0.136	<0.0001	22.43	22.90	23.64	0.034	<0.0001	1.28	1.25	1.24	<0.001	<0.0001
14	94.17	98.53	99.83	<0.0001	<0.0001	25.65	26.95	27.34	<0.0001	<0.0001	1.26	1.24	1.21	0.0607	<0.0001
15	106.28	111.94	112.69	<0.0001	<0.0001	29.04	30.74	30.99	<0.0001	<0.0001	1.24	1.22	1.19	0.114	<0.0001
16	119.39	124.62	125.63	0.0001	<0.0001	32.55	34.19	34.53	<0.0001	<0.0001	1.22	1.20	1.17	0.032	<0.0001
17	133.11	136.61	138.53	0.016	<0.0001	36.12	37.33	37.94	0.002	<0.0001	1.20	1.18	1.16	0.001	<0.0001
18	146.86	148.17	151.31	0.412	<0.001	39.65	40.27	41.24	0.146	<0.0001	1.19	1.15	1.16	<0.0001	<0.001
19	160.18	159.72	163.93	0.789	0.007	43.08	43.20	44.50	0.799	0.001	1.18	1.14	1.15	<0.0001	0.005
20	172.91	171.41	176.35	0.423	0.021	46.38	46.19	47.72	0.704	0.003	1.17	1.12	1.15	<0.0001	0.026
21	184.95	183.16	188.47	0.367	0.021	49.52	49.21	50.90	0.553	0.003	1.16	1.11	1.14	<0.0001	0.069
22	196.25	194.9	200.23	0.514	0.010	52.51	52.25	54.02	0.642	0.001	1.15	1.10	1.14	<0.0001	0.141
23	206.85	206.54	211.59	0.887	0.003	55.34	55.30	57.06	0.940	<0.001	1.14	1.10	1.13	<0.0001	0.252
24	216.83	218.01	222.51	0.614	<0.001	58.04	58.33	60.03	0.650	0.0001	1.14	1.10	1.13	<0.0001	0.406
25	226.31	229.22	233.01	0.250	<0.001	60.64	61.33	62.91	0.322	<0.0001	1.13	1.09	1.13	<0.0001	0.582
26	235.48	240.11	243.12	0.089	0.0001	63.19	64.28	65.70	0.149	<0.0001	1.12	1.09	1.12	<0.0001	0.751
27	244.58	250.59	252.88	0.036	<0.0001	65.73	67.16	68.43	0.076	<0.0001	1.11	1.08	1.11	0.0001	0.905
28	253.86	260.60	262.37	0.022	<0.0001	68.34	69.94	71.08	0.052	<0.0001	1.10	1.08	1.10	<0.001	0.937
29	263.44	270.09	271.68	0.026	<0.0001	71.04	72.62	73.69	0.057	<0.0001	1.09	1.07	1.09	0.001	0.770
30	273.10	279.01	280.74	0.056	<0.0001	73.77	75.18	76.23	0.100	<0.0001	1.08	1.06	1.08	0.002	0.634
31	282.54	287.36	289.40	0.143	<0.001	76.45	77.60	78.66	0.214	<0.001	1.06	1.05	1.07	0.016	0.577
32	291.43	295.10	297.48	0.298	0.008	78.99	79.87	80.97	0.384	0.004	1.05	1.04	1.05	0.088	0.589
33	299.38	302.26	304.79	0.441	0.027	81.28	81.98	83.11	0.572	0.015	1.03	1.02	1.04	0.270	0.633
34	306.02	308.83	311.15	0.462	0.031	83.23	83.93	85.04	0.523	0.013	1.02	1.01	1.02	0.621	0.706
35	311.07	314.80	316.38	0.336	0.020	84.74	85.70	86.73	0.387	0.004	1.00	1.00	1.00	0.764	0.850
36	314.81	319.88	320.52	0.216	0.027	85.89	87.27	88.20	0.246	0.004	0.99	0.99	0.98	0.384	0.950
37	317.72	323.72	323.74	0.174	0.045	86.76	88.62	89.47	0.159	0.003	0.97	0.98	0.97	0.374	0.786
38	320.28	325.94	326.21	0.216	0.049	87.48	89.70	90.61	0.103	0.001	0.95	0.96	0.95	0.700	0.618
39	323.00	326.17	328.14	0.500	0.127	88.15	90.50	91.65	0.099	0.001	0.94	0.93	0.93	0.321	0.560
40	326.42	324.09	329.75	0.702	0.628	88.91	90.99	92.66	0.314	0.074	0.92	0.89	0.91	0.064	0.750

HC/AC ratio, head circumference/abdominal circumference ratio.

## Data Availability

Data described in the manuscript, code book and analytic code will be available upon request pending application and approval of a data-sharing agreement.

## Supplementary Materials

The following supporting information can be downloaded at https://www.mdpi.com/article/10.3390/nu15153287/s1; Supplementary Table S1. Median and IQR of SFAs; Supplementary Table S2. Numbers in different ultrasound schedule groups among 321 subjects; Supplementary Table S3. Maternal characteristics according to weighting; Supplementary Table S4A. Association between individual SFAs and fetal growth biometrics (EFW, AC and FL) throughout 10–40 weeks of gestation; Supplementary Table S4B. Association between individual SFAs and fetal growth biometrics (HC, BPD and HC/AC ratio) throughout 10–40 weeks of gestation; Supplementary Table S5A. Back-transformed and pairwise comparison of weekly fetal growth biometrics across myristic acid (14:0) tertiles in the NICHD Fetal Growth Studies—Singletons cohort; Supplementary Table S5B. Back-transformed and pairwise comparison of weekly fetal growth biometrics across myristic acid (14:0) tertiles in the NICHD Fetal Growth Studies—Singletons cohort; Supplementary Table S6. Back-transformed and pairwise ratio of weekly fetal growth biometrics across palmitic acid (16:0) tertiles in the NICHD Fetal Growth Studies—Singletons cohort; Supplementary Table S7A. Back-transformed and pairwise comparison of weekly fetal growth biometrics across sum of even-chain SFA tertiles in the NICHD Fetal Growth Studies—Singletons cohort; Supplementary Table S7B. Back-transformed and pairwise comparison of weekly fetal growth biometrics across sum of even-chain saturated fatty acid tertiles in the NICHD Fetal Growth Studies—Singletons cohort; Supplementary Table S8A. Back-transformed and pairwise comparison of weekly fetal growth biometrics across Heptadecanoic acid (17:0) tertiles in the NICHD Fetal Growth Studies—Singletons cohort; Supplementary Table S8B. Back-transformed and pairwise comparison of weekly fetal growth biometrics across Heptadecanoic acid (17:0) tertiles in the NICHD Fetal Growth Studies—Singletons cohort; Supplementary Table S9A. Back-transformed and pairwise comparison of weekly fetal growth biometrics across sum of odd-chain saturated fatty acids tertiles in the NICHD Fetal Growth Studies—Singletons cohort; Supplementary Table S9B. Back-transformed and pairwise comparison of weekly fetal growth biometrics across sum of odd-chain saturated fatty acid tertiles in the NICHD Fetal Growth Studies—Singletons cohort; Supplementary Table S10A. Back-transformed and pairwise comparison of weekly fetal growth biometrics across lignoceric acid (24:0) tertiles in the NICHD Fetal Growth Studies—Singletons cohort; Supplementary Table S10B. Back-transformed and pairwise comparison of weekly fetal growth biometrics across lignoceric acid (24:0) tertiles in the NICHD Fetal Growth Studies—Singletons cohort; Supplementary Table S11. Back-transformed and pairwise comparison of weekly fetal growth biometrics across arachidic acid (20:0) tertiles in the NICHD Fetal Growth Studies—Singletons cohort; Supplementary Table S12A. Back-transformed and pairwise comparison of weekly fetal growth biometrics across Behenic acid (22:0) tertiles in the NICHD Fetal Growth Studies—Singletons cohort; Supplementary Table S12B. Back-transformed and pairwise comparison of weekly fetal growth biometrics across Behenic acid (22:0) tertiles in the NICHD Fetal Growth Studies—Singletons cohort; Supplementary Table S13A. Back-transformed and pairwise comparison of weekly fetal growth biometrics across sum of very-long-even-chain saturated fatty acids tertiles in the NICHD Fetal Growth Studies—Singletons cohort; Supplementary Table S13B. Back-transformed and pairwise comparison of weekly fetal growth biometrics across sum of very long even-chain saturated fatty acid tertiles in the NICHD Fetal Growth Studies—Singletons cohort; Supplementary Table S14. Association between individual SFAs and fetal growth throughout 10–40 weeks of gestation using cubic spline model with gestational weeks after adjusting for maternal family history of diabetes; Supplementary Table S15. Association between individual SFAs and fetal growth throughout 10–40 weeks of gestation using cubic spline model with gestational weeks after adjusting for maternal random glucose level at visit 0; Supplementary Table S16. Association between individual SFAs and fetal growth throughout 10–40 weeks of gestation using cubic spline model with gestational weeks after adjusting for maternal total cholesterol level at visit 0; Table S17: Association between individual SFA and fetal growth throughout between 10–40 weeks gestation using cubic spline model with gestational weeks after adjusting for subgroup SFAs at visit 0. Supplementary Figure S1. Study flow chart; Supplementary Figure S2. Back-transformed geometric means of estimated fetal growth, abdominal circumference, head circumference and biparietal diameter by gestational weeks for tertiles of myristic acid (14:0) and stearic acid (18:0), respectively, within the NICHD Fetal Growth Studies-Singletons cohort, 10–40 weeks of gestational age. The 1st (lowest) tertile curve is in blue, the 2nd (middle) tertile curve is in red, and the 3rd (highest) tertile is in green; Supplementary Figure S3. Back-transformed geometric means of femur length and gestational weeks for tertiles of the sum of even-chain SFAs within the NICHD Fetal Growth Studies—Singletons cohort, 10–40 weeks of gestational age. The 1st (lowest) tertile curve is in blue, the 2nd (middle) tertile curve is in red, and the 3rd (highest) tertile is in green. The gray shaded area and the blown-up graph on the right indicate a significant decrement in femur length from 14 to 18 weeks of gestation in both the 2nd and 3rd tertiles, compared with the 1st tertile; Supplementary Figure S4. Back-transformed geometric means of abdominal circumference and head circumference by gestational weeks for tertiles of lignoceric acid (24:0) and very long even-chain SFAs, respectively, within the NICHD Fetal Growth Studies—Singletons cohort, 10–40 weeks of gestational age. The 1st (lowest) tertile curve is in blue, the 2nd (middle) tertile curve is in red, and the 3rd (highest) tertile is in green. The gray shaded area and the blown-up graph on the right indicate a significant decrement in abdominal circumference from 29 to 39 week of gestation (Figure S4A) in both the 2nd and 3rd tertiles of lignoceric acid (24:0), compared with the 1st tertile. The gray shaded area and the blown-up graph on the right indicate a significant decrement in head circumference from 18 to 33 week of gestation (Figure S4B) in both the 2nd and 3rd tertiles of very long even-chain SFAs, compared with the 1st tertile.
