# Supplementary material for: Early Pregnancy Maternal Plasma Phospholipid Saturated Fatty Acids and Fetal Growth: Findings from a Multi-Racial/Ethnic Birth Cohort in US
Source: Nutrients. 2023 Jul 25;15(15):3287. doi: 10.3390/nu15153287 (PMC10420908; doi:10.3390/nu15153287)
Supplement: Supplementary file 1 [file nutrients-15-03287-s001.zip › nutrients-2462325-supplementary-figure.pdf]

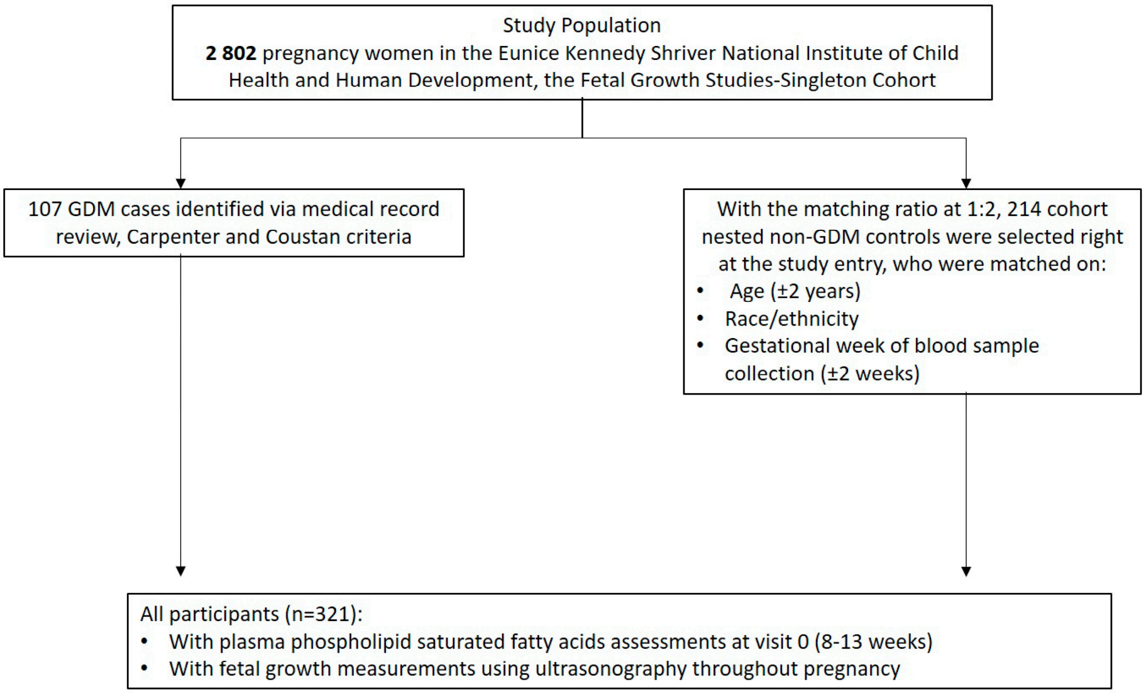

Figure S1. Study flow chart.

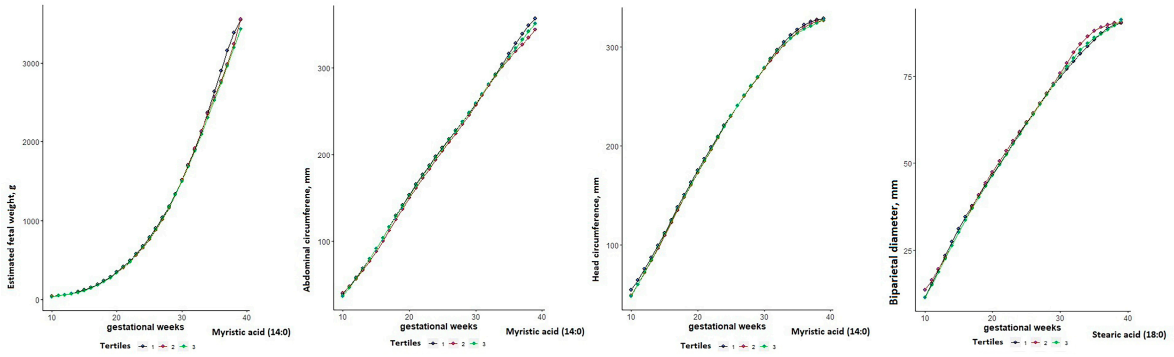

Figure S2. Back-transformed geometric means of estimated fetal growth, abdominal circumference, head circumference and biparietal diameter by gestational weeks for tertiles of myristic acid (14:0) and stearic acid (18:0), respectively, within the NICHD Fetal Growth Studies-Singletons cohort, 10–40 weeks of gestational age. The 1st (lowest) tertile curve is in blue, the 2nd (middle) tertile curve is in red, and the 3rd (highest) tertile is in green;.

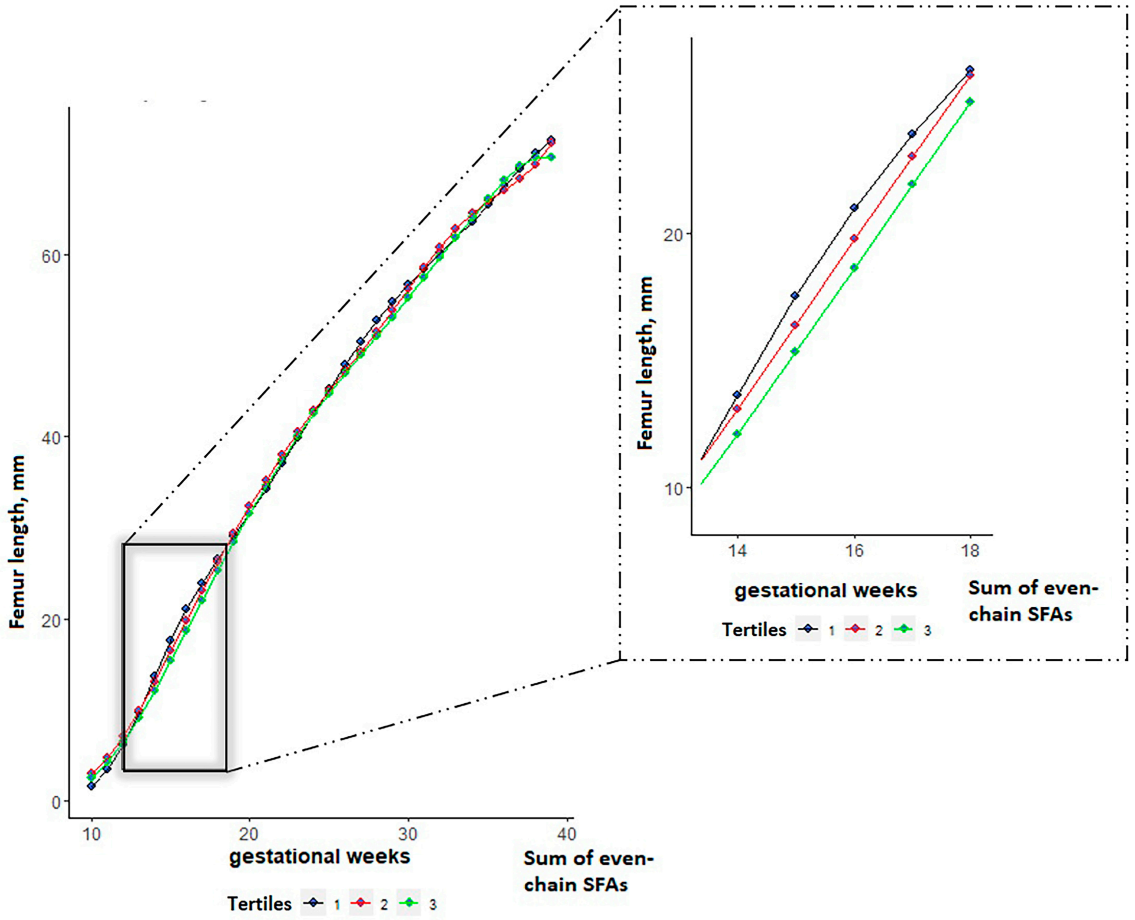

Figure S3. Back-transformed geometric means of femur length and gestational weeks for tertiles of the sum of even-chain SFAs within the NICHD Fetal Growth Studies—Singletons cohort, 10–40 weeks of gestational age. The 1st (lowest) tertile curve is in blue, the 2nd (middle) tertile curve is in red, and the 3rd (highest) tertile is in green. The gray shaded area and the blown-up graph on the right indicate a significant decrement in femur length from 14 to 18 weeks of gestation in both the 2nd and 3rd tertiles, compared with the 1st tertile.

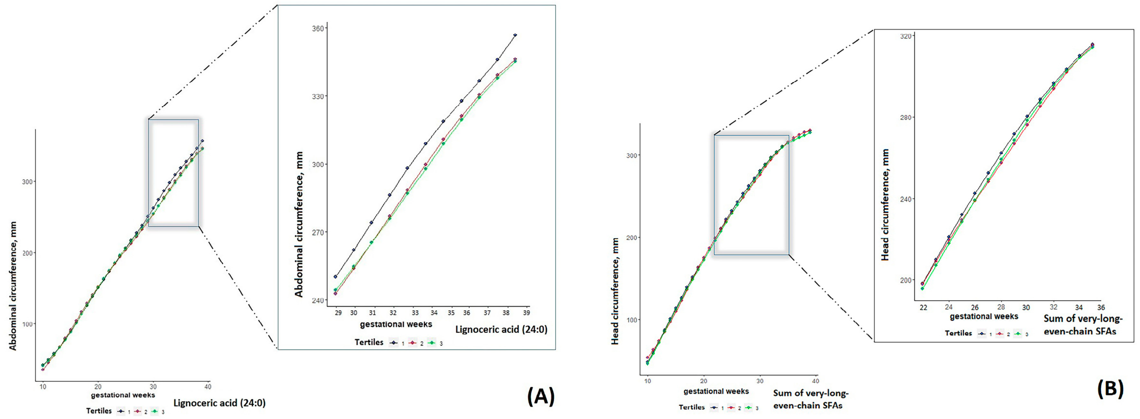

Figure S4. Back-transformed geometric means of abdominal circumference and head circumference by gestational weeks for tertiles of lignoceric acid (24:0) and very long even-chain SFAs, respectively, within the NICHD Fetal Growth Studies—Singletons cohort, 10–40 weeks of gestational age. The 1st (lowest) tertile curve is in blue, the 2nd (middle) tertile curve is in red, and the 3rd (highest) tertile is in green. The gray shaded area and the blown-up graph on

the right indicate a significant decrement in abdominal circumference from 29 to 39 week of gestation (Figure S4A) in both the 2nd and 3rd tertiles of lignoceric acid (24:0), compared with the 1st tertile. The gray shaded area and the blown-up graph on the right indicate a significant decrement in head circumference from 18 to 33 week of gestation (Figure S4B) in both the 2nd and 3rd tertiles of very long even-chain SFAs, compared with the 1st tertile.
