# Supplementary material for: Early Pregnancy Maternal Plasma Phospholipid Saturated Fatty Acids and Fetal Growth: Findings from a Multi-Racial/Ethnic Birth Cohort in US
Source: Nutrients. 2023 Jul 25;15(15):3287. doi: 10.3390/nu15153287 (PMC10420908; doi:10.3390/nu15153287)
Supplement: Supplementary file 1 [file nutrients-15-03287-s001.zip › nutrients-2462325-supplementary-table.pdf]

Supplementary Table S1. Individual fatty acid in weight percentage (%) of the total plasma phospholipid FA fraction

Fatty acids	% of total plasma phospholipid FAs<br>Mean, SD
SFAs	
myristic acid (14:0)	0.31 ± 0.08
pentadecanoic acid (15:0)	0.25 ± 0.07
palmitic acid (16:0)	27.45 ± 1.62
heptadecanoic acid (17:0)	0.43 ± 0.73
stearic acid (18:0)	12.72 ± 1.21
eicosanoic acid (20:0)	0.29 ± 0.10
docosanoic acid (22:0)	0.74 ± 0.32
tetracosanoic acid (24:0)	0.56 ± 0.20
MUFAs	
palmitoleic acid (16:1n7)	0.54 ± 0.20
petroselinic acid (18:1n6)	0.20 ± 0.08
vacenic acid (18:1n7)	1.38 ± 0.20
oleic acid (18:1n9)	7.34 ± 0.90
gondoic acid (20:1n9)	0.14 ± 0.07
vervonic acid (24:1n9)	1.04 ± 0.47
PUFAs	
alpha-linolenic acid (ALA; 18:3n3)	0.22 ± 0.07
eicosapentaenoic acid (EPA; 20:5n3)	0.33 ± 0.18
n-3 docosapentaenoic acid (DPA; 22:5n3)	0.69 ± 0.21
docosahexaenoic acid (DHA; 22:6n3)	4.24 ± 1.25
linolenic acid (LA;18:2n6)	20.40 ± 2.73
gamma-linolenic acid (GLA; 18:3n6)	0.08 ± 0.03
eicosadienoic acid (EDA; 20:2n6)	0.51 ± 0.10
dihomo-gamma-linolenic acid (DGLA; 20:3n6)	3.58 ± 0.92
arachidonic acid (AA; 20:4n6)	11.21 ± 2.08
docosatetraenoic acid (DTA; 22:4n6)	0.49 ± 0.20
n-6 docosapentaenoic acid (n-6 DPA; 22:5n6)	0.54 ± 0.21

Supplementary Table S2. Numbers in different ultrasound schedule groups among 321 subjects

Ultrasound schedule,	GDM group	Non-GDM group	Total number	Total
Group A	28	56	84	
Group B	35	70	105	
Group C	24	48	72	
Group D	20	40	60	321

Supplementary Table S3. Maternal characteristics according to weighting

Maternal characteristics	Overall (n=2802)	Fetal growth study (n=321)	
		Non-weighted	Weighted
	n (%)	n (%)	Mean percentage (95% CI)
Age, years			
<25	805 (28.7%)	54 (16.8%)	30.0 (21.6, 38.4)%
25-29	821 (29.3%)	85 (26.5%)	29.7 (22.7, 36.7)%
30-34	788 (28.1%)	97 (30.2%)	25.8 (19.6, 31.9)%
≥ 35	388 (13.8%)	85 (26.5%)	14.5 (10.5, 18.5)%
Race/ethnicity			%
Non-Hispanic White	751 (26.8)	75 (23.4%)	30.9 (23.3, 38.6)%
Non-Hispanic Black	781 (27.9)	45 (14.0%)	23.3 (15.6, 31.0)%
Hispanic	803 (28.7)	123 (38.3%)	27.2 (21.2, 33.2)%
Asian/Pacific Islander	467 (16.7)	78 (24.3%)	18.5 (13.4, 23.7)%
Pre-pregnancy BMI, kg/m^2			
<25.0	1567 (55.9%)	156 (48.6%)	51.7 (43.9, 59.6)%
25.0-29.9	767 (27.4%)	99 (30.8%)	33.1 (25.3, 40.8)%
≥ 30	453 (16.2%)	64 (19.9%)	15.0 (9.83, 20.15)%
Education			
Missing	839 (29.9)	81 (25.2%)	25.1 (17.8, 32.4)%
Below college	850 (30.3)	117 (36.4%)	35.2 (27.7, 42.7)%
College degree and above	1111 (39.7)	123 (38.3%)	39.8 (32.3, 47.3)%
Family history of diabetes, Yes	590 (21.1%)	88 (27.4%)	20.4 (14.6, 26.3)%
Married or co-habiting, Yes	2082 (74.3%)	259 (80.7%)	72.87 (65.18, 80.56)%
Nulliparous, Yes	1319 (47.1)	144 (44.9%)	51.1 (43.3, 58.9)%
GDM diagnosis, Yes	107 (3.8)	107 (33.3%)	3.9 (2.9, 4.8)%
Gestational weeks at delivery			
<37 weeks	159 (5.7%)	22 (6.9%)	9.12 (3.61, 14.6)%
37-39 weeks	1540 (55.0%)	204 (63.6%)	56.1 (48.2, 64.1)%
≥ 40 weeks	858 (30.6%)	93 (29.0%)	34.7 (27.1, 42.4)%
Fetal sex, Male	1309 (46.7%)	166 (51.7%)	51.5 (43.6, 59.4)%
Birth weight, g			
≤ 2500	131 (4.7%)	14 (4.4%)	6.75 (1.76, 11.7)%
2501-3999	2228 (79.5%)	274 (85.4%)	87.2 (81.5, 92.9)%
≥ 4000	192 (6.9%)	30 (9.3%)	6.03 (2.79, 9.26)%
SGA, Yes	221 (7.9%)	13 (4.0%)	4.65 (1.94, 7.36)%
LGA, Yes	223 (8.0%)	34 (10.6%)	7.13 (3.38, 10.9)%
Delivery mode,			
Vaginal delivery	1810 (64.6%)	203 (63.2%)	62.4 (54.5, 70.3)%
C-section	750 (26.8%)	116 (36.1%)	37.6 (29.7, 45.5)%

Abbreviations: BMI, Body mass index; GDM, gestational diabetes mellitus; SGA, smaller than gestational age; LGA, larger than gestational age.

Supplementary Table S4A. Association between individual SFA and fetal growth biometrics (EFW, AC & FL) throughout between 10-40 weeks gestation

SFAs in tertiles	Estimated fetal growth (EFW), g				Abdominal circumference (AC), mm				Femur length (FL), mm			
	Global test after adjusting for maternal age, ethnicity/race, nulliparity, pre-pregnancy body mass indes and fetal sex				Global test after adjusting for maternal age, ethnicity/race, nulliparity, pre-pregnancy body mass indes and fetal sex				Global test after adjusting for maternal age, ethnicity/race, nulliparity, pre-pregnancy body mass indes and fetal sex			
	Without interaction term SFA*GA		With additional interaction SFA*GA		Without interaction term SFA*GA		With additional interaction SFA*GA		Without interaction term PUFA*GA		With additional interaction SFA*GA	
			likelihood ratio	Bonferroni correction			likelihood ratio	Bonferroni correction			likelihood ratio	Bonferroni correction
	Mean difference in log (SE)		P value	P value	Mean difference in log (SE)		P value	P value	Mean difference in log (SE)		P value	P value
Myristic acid (14:0)	1st tertile	Reference	**<0.00001**	**<0.00001**	1st tertile	Reference	**<0.00001**	**<0.00001**	1st tertile	Reference	**<0.001**	**0.012**
	2nd tertile	-0.0221 (0.0222)			2nd tertile	-0.0224 (0.0040)			2nd tertile	-0.0152 (0.0201)		
	3rd tertile	-0.0269 (0.0236)			3rd tertile	-0.0252 (0.0042)			3rd tertile	-0.0202 (0.0213)		
Pentadecanoic acid (15:0)	1st tertile	Reference	**<0.00001**	**<0.00001**	1st tertile	Reference	**<0.00001**	**<0.00001**	1st tertile	Reference	**<0.00001**	**0.005**
	2nd tertile	0.0149 (0.0224)			2nd tertile	-0.0067 (0.0044)			2nd tertile	0.0109 (0.0206)		
	3rd tertile	0.02937 (0.0225)			3rd tertile	-0.0036 (0.0040)			3rd tertile	0.0135 (0.0207)		
Palmitic acid (16:0)	1st tertile	Reference	**0.003**	0.207	1st tertile	Reference	**<0.001**	**0.020**	1st tertile	Reference	0.399	1.00
	2nd tertile	-0.0100 (0.0226)			2nd tertile	0.0024 (0.0041)			2nd tertile	-0.0030 (0.0205)		
	3rd tertile	-0.0273 (0.0229)			3rd tertile	0.0046 (0.0042)			3rd tertile	-0.0175 (0.0208)		
Heptadecanoic acid (17:0)	1st tertile	Reference	**<0.00001**	**<0.00001**	1st tertile	Reference	**<0.00001**	**<0.001**	1st tertile	Reference	**<0.00001**	**<0.00001**
	2nd tertile	-0.0369 (0.0216)			2nd tertile	-0.0220 (0.0040)			2nd tertile	-0.0093 (0.0197)		
	3rd tertile	0.0001 (0.0225)			3rd tertile	0.0010 (0.0040)			3rd tertile	0.0039 (0.0206)		
Stearic acid (18:0)	1st tertile	Reference	**<0.00001**	**<0.00001**	1st tertile	Reference	**<0.00001**	**<0.00001**	1st tertile	Reference	**<0.00001**	**<0.00001**
	2nd tertile	-0.0221 (0.0219)			2nd tertile	-0.0123 (0.0042)			2nd tertile	-0.0158 (0.0199)		
	3rd tertile	-0.0146 (0.0225)			3rd tertile	-0.0195 (0.0042)			3rd tertile	-0.0044 (0.0205)		
Arachidic acid (20:0)	1st tertile	Reference	**0.0019**	0.124	1st tertile	Reference	**0.012**	0.790	1st tertile	Reference	**<0.00001**	**<0.001**
	2nd tertile	-0.0249 (0.0214)			2nd tertile	0.0027 (0.0044)			2nd tertile	-0.0199 (0.0194)		
	3rd tertile	-0.0410 (0.0239)			3rd tertile	-0.0139 (0.0042)			3rd tertile	-0.0221 (0.0219)		

Behenic acid (22:0)	1st tertile	Reference	**<0.00001**	**<0.00001**	1st tertile	Reference	**<0.00001**	**<0.00001**	1st tertile	Reference	**0.004**	0.230
	2nd tertile	-0.0350 (0.0219)			2nd tertile	0.0108 (0.0044)			2nd tertile	-0.0184 (0.0199)		
	3rd tertile	-0.0373 (0.0231)			3rd tertile	-0.0174 (0.0042)			3rd tertile	-0.0280 (0.0212)		
Lignoceric acid (24:0)	1st tertile	Reference	**<0.00001**	**<0.00001**	1st tertile	Reference	**<0.00001**	**<0.00001**	1st tertile	Reference	**<0.001**	**0.028**
	2nd tertile	0.0099 (0.0218)			2nd tertile	-0.0107 (0.0043)			2nd tertile	0.0010 (0.0198)		
	3rd tertile	-0.0016 (0.0230)			3rd tertile	-0.0140 (0.0043)			3rd tertile	-0.0032 (0.0209)		
Sum of even-chain SFAs (14:0 + 16:0 + 18:0)	1st tertile	Reference	**<0.00001**	**<0.00001**	1st tertile	Reference	**<0.00001**	**<0.00001**	1st tertile	Reference	**<0.00001**	**<0.00001**
	2nd tertile	0.0059 (0.0219)			2nd tertile	0.0113 (0.0040)			2nd tertile	0.0004 (0.0199)		
	3rd tertile	-0.0384 (0.0232)			3rd tertile	-0.0057 (0.0042)			3rd tertile	-0.0217 (0.0211)		
Sum of odd-chain SFAs (15:0 + 17:0)	1st tertile	Reference	**<0.00001**	**<0.00001**	1st tertile	Reference	0.112	1.00	1st tertile	Reference	**<0.00001**	**0.004**
	2nd tertile	-0.0237 (0.0225)			2nd tertile	-0.0066 (0.0041)			2nd tertile	-0.0025 (0.0206)		
	3rd tertile	0.0205 (0.0224)			3rd tertile	0.0017 (0.0040)			3rd tertile	0.0193 (0.0204)		
Sum of very-long-even-chain SFAs (20:0 + 22:0 + 24:0)	1st tertile	Reference	**<0.00001**	**<0.00001**	1st tertile	Reference	**<0.00001**	**<0.00001**	1st tertile	Reference	**0.001**	0.064
	2nd tertile	-0.0498 (0.0215)			2nd tertile	-0.0016 (0.0044)			2nd tertile	-0.0268 (0.0194)		
	3rd tertile	-0.0335 (0.0235)			3rd tertile	-0.0227 (0.0042)			3rd tertile	-0.0212 (0.0215)		

Supplementary Table S4B. Association between individual SFA and fetal growth biometrics (HC, BPD, HC/AC ratio) throughout between 10-40 weeks gestation

SFAs in tertiles	Head circumference (HC), mm				Biparietal diameter (BPD), mm				HC/AC ratio			
	Global test after adjusting for maternal age, ethnicity/race, nulliparity, pre-pregnancy body mass indes and fetal sex				Global test after adjusting for maternal age, ethnicity/race, nulliparity, pre-pregnancy body mass indes and fetal sex				Global test after adjusting for maternal age, ethnicity/race, nulliparity, pre-pregnancy body mass indes and fetal sex			
	Without interaction term SFA*GA		With additional interaction SFA*GA		Without interaction term SFA*GA		With additional interaction SFA*GA		Without interaction term SFA*GA		With additional interaction SFA*GA	
			likelihood ratio	Bonferroni correction			likelihood ratio	Bonferroni correction			likelihood ratio	Bonferroni correction
	Mean difference in log (SE)		P value	P value	Mean difference in log (SE)		P value	P value	Mean difference in log (SE)		P value	P value
14:0	1st tertile	Reference	**<0.00001**	**<0.00001**	1st tertile	Reference	**<0.00001**	**<0.00001**	1st tertile	Reference	**<0.00001**	**<0.00001**
	2nd tertile	-0.0135 (0.0033)			2nd tertile	-0.0166 (0.0038)			2nd tertile	0.0094 (0.0030)		
	3rd tertile	-0.0267 (0.0034)			3rd tertile	-0.0239 (0.0039)			3rd tertile	-0.0011 (0.0032)		
15:0	1st tertile	Reference	**<0.00001**	**<0.00001**	1st tertile	Reference	**<0.00001**	**<0.00001**	1st tertile	Reference	**<0.00001**	**<0.00001**
	2nd tertile	0.0006 (0.0136)			2nd tertile	0.0017 (0.0041)			2nd tertile	0.0037 (0.0033)		
	3rd tertile	0.0112 (0.0137)			3rd tertile	0.0037 (0.0037)			3rd tertile	-0.0012 (0.0030)		
16:0	1st tertile	Reference	0.717	1.00	1st tertile	Reference	**0.025**	1.00	1st tertile	Reference	**<0.00001**	**<0.00001**
	2nd tertile	0.0003 (0.0139)			2nd tertile	-0.0007 (0.0038)			2nd tertile	-0.0044 (0.0031)		
	3rd tertile	-0.0054 (0.0140)			3rd tertile	-0.0003 (0.0039)			3rd tertile	-0.0103 (0.0031)		
17:0	1st tertile	Reference	**<0.00001**	**<0.00001**	1st tertile	Reference	**<0.00001**	**<0.00001**	1st tertile	Reference	**0.028**	1.00
	2nd tertile	-0.0256 (0.0032)			2nd tertile	-0.0218 (0.0037)			2nd tertile	-0.0038 (0.0030)		
	3rd tertile	0.0021 (0.0032)			3rd tertile	0.0066 (0.0037)			3rd tertile	0.0016 (0.0030)		
18:0	1st tertile	Reference	**<0.00001**	**<0.00001**	1st tertile	Reference	**<0.00001**	**<0.00001**	1st tertile	Reference	**<0.00001**	**<0.00001**
	2nd tertile	0.0084 (0.0034)			2nd tertile	0.0132 (0.0039)			2nd tertile	0.0208 (0.0031)		
	3rd tertile	0.0030 (0.0035)			3rd tertile	0.0023 (0.0039)			3rd tertile	0.0225 (0.0031)		
20:0	1st tertile	Reference	**<0.00001**	**0.001**	1st tertile	Reference	**<0.00001**	**<0.00001**	1st tertile	Reference	**<0.001**	**0.017**
	2nd tertile	-0.0025 (0.0036)			2nd tertile	-0.0025 (0.0041)			2nd tertile	-0.0057 (0.0033)		
	3rd tertile	-0.0188 (0.0034)			3rd tertile	-0.0192 (0.0039)			3rd tertile	-0.0052 (0.0031)		
22:0	1st tertile	Reference	**<0.00001**	**<0.00001**	1st tertile	Reference	**<0.00001**	**<0.00001**	1st tertile	Reference	**<0.00001**	**<0.00001**
	2nd tertile	2.97E-06 (0.0036)			2nd tertile	-0.0012 (0.0041)			2nd tertile	-0.0112 (0.0033)		
	3rd tertile	-0.0201 (0.0035)			3rd tertile	-0.0216 (0.0039)			3rd tertile	-0.0030 (0.0032)		
24:0	1st tertile	Reference	**<0.00001**	**<0.00001**	1st tertile	Reference	**<0.00001**	**<0.00001**	1st tertile	Reference	**<0.00001**	**<0.00001**
	2nd tertile	-0.0250 (0.0035)			2nd tertile	-0.0321 (0.0039)			2nd tertile	-0.0153 (0.0031)		
	3rd tertile	-0.0075 (0.0035)			3rd tertile	-0.0170 (0.0039)			3rd tertile	0.0049 (0.0032)		
Sum of even-chain	1st tertile	Reference	**<0.00001**	**<0.00001**	1st tertile	Reference	**<0.00001**	**<0.00001**	1st tertile	Reference	**<0.00001**	**<0.00001**
SFAs	2nd tertile	0.0100 (0.0032)			2nd tertile	0.0134 (0.0037)			2nd tertile	-0.0009 (0.0030)		
(14:0 + 16:0 + 18:0)	3rd tertile	-0.0132 (0.0034)			3rd tertile	-0.0093 (0.0039)			3rd tertile	-0.0066 (0.0032)		
Sum of odd-chain SFAs	1st tertile	Reference	**<0.00001**	**<0.00001**	1st tertile	Reference	**<0.00001**	**<0.00001**	1st tertile	Reference	**0.002**	0.117
(15:0 + 17:0)	2nd tertile	-0.0101 (0.0034)			2nd tertile	-0.0077 (0.0038)			2nd tertile	-0.0041 (0.0031)		
	3rd tertile	0.0043 (0.0033)			3rd tertile	0.0108 (0.0037)			3rd tertile	0.0031 (0.0030)		
Sum of very-long-	1st tertile	Reference	**<0.00001**	**<0.00001**	1st tertile	Reference	**<0.00001**	**<0.00001**	1st tertile	Reference	**<0.00001**	**<0.00001**
even-chain SFAs	2nd tertile	-0.0091 (0.0036)			2nd tertile	-0.0124 (0.0041)			2nd tertile	-0.0076 (0.0033)		
(20:0 + 22:0 + 24:0)	3rd tertile	-0.0020 (0.0035)			3rd tertile	-0.0245 (0.0039)			3rd tertile	0.0008 (0.0032)		

Supplementary Table S5A. Back-transformed and pairwise comparison of weekly fetal growth biometrics across myristic acid (14:0) tertiles in the NICHD Fetal Growth Studies-Singletons

GW	Estimated fetal weight (EFW), g					Abdominal circumference (AC), mm					Femur length (FL), mm				
	Back-transformed geometric mean gram			Wald test for pairwise comparison p value		Back-transformed geometric mean mm			Wald test for pairwise comparison p value		Back-transformed geometric mean mm			Wald test for pairwise comparison p value	
	1st tertile	2nd tertile	3rd tertile	2nd vs 1st tertile	3rd vs 1st tertile	1st tertile	2nd tertile	3rd tertile	2nd vs 1st tertile	3rd vs 1st tertile	1st tertile	2nd tertile	3rd tertile	2nd vs 1st tertile	3rd vs 1st tertile
10	37.47	38.76	33.86	0.711	0.241	39.67	40.25	36.11	0.785	**0.037**	2.61	2.30	2.41	0.112	0.278
11	45.81	46.22	43.51	0.852	0.258	48.21	48.10	45.95	0.937	**0.052**	4.43	4.02	4.11	**0.025**	0.056
12	56.84	56.27	55.86	0.652	0.431	57.73	56.92	56.73	0.280	0.217	6.87	6.38	6.40	**0.002**	**0.001**
13	71.32	69.63	71.59	0.183	0.830	68.16	66.69	68.16	**0.040**	0.994	9.82	9.29	9.24	**0.009**	**0.001**
14	90.09	87.16	91.41	0.130	0.479	79.38	77.33	79.95	**0.043**	0.608	13.10	12.58	12.45	0.081	**0.017**
15	114.13	109.85	116.11	0.096	0.412	91.23	88.73	91.86	**0.040**	0.630	16.50	16.02	15.85	0.207	0.064
16	144.40	138.74	146.52	0.061	0.455	103.54	100.75	103.70	**0.030**	0.910	19.84	19.39	19.26	0.309	0.149
17	181.71	174.76	183.37	**0.044**	0.619	116.11	113.17	115.39	**0.025**	0.637	23.01	22.58	22.56	0.367	0.310
18	226.52	218.53	227.28	0.057	0.859	128.70	125.73	126.94	**0.037**	0.300	26.05	25.59	25.75	0.384	0.557
19	278.82	270.03	278.63	0.095	0.972	141.12	138.17	138.46	0.062	0.161	29.05	28.53	28.90	0.379	0.789
20	338.92	329.27	337.98	0.130	0.886	153.23	150.30	149.90	0.086	0.110	32.01	31.47	31.97	0.407	0.953
21	407.24	396.66	405.89	0.153	0.859	164.92	162.03	161.21	0.103	0.095	34.89	34.37	34.93	0.466	0.946
22	484.19	472.64	482.91	0.169	0.881	176.15	173.33	172.34	0.117	0.103	37.65	37.22	37.74	0.562	0.897
23	570.18	557.74	569.55	0.195	0.948	186.93	184.19	183.28	0.142	0.139	40.28	39.98	40.38	0.702	0.891
24	665.69	652.59	666.34	0.248	0.954	197.28	194.68	194.03	0.191	0.216	42.77	42.64	42.85	0.874	0.921
25	771.31	758.04	773.79	0.333	0.854	207.31	204.91	204.64	0.269	0.346	45.13	45.19	45.16	0.947	0.972
26	887.78	875.21	892.49	0.445	0.771	217.16	215.02	215.18	0.368	0.517	47.37	47.63	47.34	0.790	0.972
27	1016.07	1005.63	1023.07	0.582	0.707	226.99	225.22	225.74	0.479	0.700	49.53	49.97	49.43	0.663	0.921
28	1157.5	1151.32	1166.31	0.763	0.664	237.03	235.74	236.45	0.607	0.863	51.63	52.23	51.49	0.567	0.884
29	1313.48	1314.54	1322.95	0.960	0.652	247.48	246.84	247.41	0.786	0.984	53.72	54.43	53.56	0.501	0.868
30	1484.95	1495.09	1493.01	0.654	0.722	258.36	258.40	258.57	0.988	0.953	55.80	56.57	55.64	0.474	0.874
31	1672.73	1690.64	1676.05	0.508	0.902	269.62	270.13	269.80	0.842	0.963	57.86	58.64	57.71	0.495	0.893
32	1877.61	1897.1	1871.22	0.562	0.846	281.20	281.71	280.96	0.865	0.954	59.88	60.63	59.75	0.550	0.908
33	2100.32	2108.42	2077.26	0.837	0.546	293.04	292.73	291.89	0.925	0.799	61.86	62.53	61.71	0.617	0.903
34	2341.57	2316.43	2292.41	0.544	0.221	305.04	302.78	302.41	0.479	0.571	63.79	64.34	63.58	0.690	0.864
35	2599.99	2512.83	2514.32	**0.039**	**0.042**	317.07	311.50	312.34	0.068	0.323	65.66	66.03	65.33	0.789	0.779
36	2864.36	2700.45	2737.9	**0.001**	**0.017**	328.76	319.41	321.91	**0.007**	0.187	67.47	67.57	66.92	0.947	0.677
37	3116.67	2889.51	2954.85	**<0.001**	**0.014**	339.64	327.44	331.91	**0.003**	0.173	69.22	68.89	68.39	0.841	0.568
38	3334.04	3093.96	3155.12	**<0.001**	**0.009**	349.17	336.60	343.25	**0.003**	0.317	70.92	69.92	69.72	0.564	0.421
39	3490.46	3331.94	3327.35	0.077	0.078	356.81	348.05	356.99	0.089	0.979	72.57	70.60	70.94	0.330	0.368
40	3559.90	3627.07	3459.62	0.718	0.631	362.00	363.09	374.37	0.917	0.288	74.18	70.87	72.06	0.346	0.555

Supplementary Table S5B. Back-transformed and pairwise comparison of weekly fetal growth biometrics across myristic acid (14:0) tertiles in the NICHD Fetal Growth Studies-Singletons

GW	Head circumference (HC), mm					Biparietal diameter (BPD), mm					HC/AC ratio				
	Back-transformed geometric mean mm			Wald test for pairwise comparison p value		Back-transformed geometric mean mm			Wald test for pairwise comparison p value		Back-transformed geometric mean			Wald test for pairwise comparison p value	
	1st tertile	2nd tertile	3rd tertile	2nd vs 1st tertile	3rd vs 1st tertile	1st tertile	2nd tertile	3rd tertile	2nd vs 1st tertile	3rd vs 1st tertile	1st tertile	2nd tertile	3rd tertile	2nd vs 1st tertile	3rd vs 1st tertile
10	55.24	48.44	47.05	**0.002**	**<0.0001**	13.35	12.27	11.77	0.077	**0.002**	1.39	1.21	1.25	**<0.001**	**0.005**
11	64.60	59.49	59.18	**<0.001**	**<0.0001**	16.41	15.49	15.41	**0.022**	**0.003**	1.34	1.24	1.25	**<0.001**	**<0.001**
12	74.83	71.38	72.17	**<0.0001**	**<0.0001**	19.68	18.98	19.32	**0.002**	0.078	1.30	1.26	1.24	**<0.001**	**<0.0001**
13	85.89	83.88	85.60	**0.006**	0.653	23.09	22.63	23.32	**0.037**	0.216	1.26	1.26	1.23	0.677	**0.002**
14	97.67	96.72	99.12	0.357	0.085	26.56	26.34	27.25	0.481	**0.007**	1.23	1.25	1.22	0.113	0.299
15	110.04	109.70	112.43	0.780	**0.015**	30.03	30.03	30.98	0.995	**0.001**	1.21	1.24	1.21	**0.018**	0.977
16	122.84	122.63	125.35	0.872	**0.0132**	33.46	33.63	34.47	0.654	**0.001**	1.19	1.22	1.19	**0.006**	0.483
17	135.86	135.42	137.85	0.727	0.057	36.81	37.12	37.73	0.432	**0.004**	1.17	1.2	1.18	**0.008**	0.263
18	148.90	148.03	150.06	0.526	0.316	40.10	40.50	40.85	0.330	**0.033**	1.16	1.18	1.17	**0.029**	0.210
19	161.71	160.54	162.17	0.432	0.722	43.35	43.82	43.96	0.281	0.122	1.15	1.16	1.16	0.085	0.203
20	174.16	172.90	174.16	0.430	0.996	46.53	47.10	47.05	0.229	0.218	1.14	1.15	1.15	0.133	0.201
21	186.16	185.04	185.95	0.496	0.885	49.64	50.30	50.12	0.171	0.266	1.13	1.14	1.14	0.137	0.201
22	197.65	196.88	197.46	0.641	0.893	52.67	53.43	53.15	0.120	0.263	1.12	1.14	1.13	0.108	0.210
23	208.63	208.37	208.64	0.877	0.995	55.60	56.46	56.12	0.087	0.235	1.12	1.13	1.13	0.074	0.247
24	219.11	219.47	219.46	0.844	0.821	58.44	59.40	59.02	0.075	0.213	1.11	1.13	1.12	0.055	0.329
25	229.15	230.17	229.9	0.606	0.653	61.22	62.25	61.84	0.078	0.212	1.11	1.12	1.11	**0.048**	0.456
26	238.84	240.49	239.97	0.443	0.536	63.93	65.02	64.57	0.086	0.236	1.10	1.12	1.11	**0.049**	0.608
27	248.31	250.45	249.66	0.341	0.475	66.61	67.74	67.22	0.092	0.287	1.10	1.11	1.10	0.054	0.764
28	257.70	260.11	259.04	0.278	0.478	69.29	70.42	69.77	0.088	0.395	1.09	1.10	1.09	0.065	0.919
29	267.15	269.52	268.12	0.255	0.586	72.00	73.09	72.24	0.079	0.645	1.08	1.09	1.08	0.105	0.915
30	276.53	278.62	276.86	0.305	0.854	74.69	75.72	74.63	0.088	0.913	1.07	1.08	1.07	0.243	0.766
31	285.67	287.27	285.19	0.469	0.802	77.31	78.26	76.94	0.152	0.515	1.06	1.06	1.06	0.520	0.696
32	294.35	295.36	293.06	0.690	0.551	79.81	80.65	79.17	0.266	0.328	1.05	1.05	1.04	0.717	0.714
33	302.33	302.73	300.39	0.884	0.402	82.11	82.82	81.34	0.381	0.272	1.03	1.03	1.03	0.644	0.801
34	309.38	309.26	307.13	0.964	0.315	84.14	84.72	83.46	0.463	0.315	1.01	1.02	1.01	0.275	0.988
35	315.28	314.83	313.20	0.856	0.332	85.85	86.30	85.53	0.550	0.626	0.99	1.01	1.00	**0.016**	0.687
36	320.03	319.52	318.38	0.853	0.510	87.25	87.57	87.45	0.701	0.793	0.97	1.00	0.98	**<0.001**	0.437
37	323.68	323.48	322.34	0.949	0.642	88.40	88.63	89.04	0.809	0.474	0.96	0.99	0.96	**<0.0001**	0.301
38	326.31	326.87	324.71	0.868	0.579	89.33	89.56	90.07	0.822	0.405	0.94	0.97	0.95	**<0.0001**	0.183
39	328.00	329.90	325.16	0.635	0.448	90.11	90.44	90.33	0.781	0.844	0.92	0.95	0.93	**0.004**	0.263
40	328.84	332.73	323.38	0.617	0.501	90.78	91.36	89.63	0.803	0.646	0.91	0.92	0.92	0.558	0.646

HC/AC ratio, head circumference/abdominal circumference ratio.

Supplementary Table S6. Back-transformed and pairwise comparison of weekly fetal growth biometrics across palmitic acid (16:0) tertiles in the NICHD Fetal Growth Studies-Singletons

GW	Abdominal circumference, mm				
	Back-transformed geometric mean, mm			Wald test p value for pairwise comparison	
	1st tertile	2nd tertile	3rd tertile	2nd vs 1st tertile	3rd vs 1st tertile
10	38.83	38.74	37.30	0.961	0.384
11	47.45	47.79	46.79	0.771	0.581
12	57.11	57.77	57.15	0.330	0.958
13	67.72	68.55	68.13	0.249	0.604
14	79.17	79.96	79.50	0.430	0.749
15	91.28	91.83	91.06	0.647	0.860
16	103.86	103.99	102.66	0.917	0.377
17	116.65	116.28	114.23	0.775	0.089
18	129.4	128.59	125.80	0.564	**0.019**
19	141.85	140.85	137.36	0.524	**0.008**
20	153.84	152.97	148.85	0.609	**0.007**
21	165.28	164.84	160.20	0.802	**0.011**
22	176.17	176.40	171.38	0.900	**0.023**
23	186.54	187.60	182.41	0.565	0.066
24	196.50	198.44	193.32	0.316	0.187
25	206.21	208.94	204.16	0.192	0.433
26	215.88	219.17	215.04	0.150	0.767
27	225.77	229.19	226.08	0.157	0.916
28	236.20	239.15	237.46	0.226	0.689
29	247.48	249.16	249.23	0.472	0.591
30	259.49	259.28	261.23	0.924	0.612
31	271.91	269.53	273.21	0.337	0.725
32	284.34	279.95	284.92	0.122	0.886
33	296.31	290.56	296.04	0.065	0.952
34	307.28	301.42	306.25	0.058	0.820
35	316.81	312.55	315.32	0.150	0.749
36	325.36	323.77	323.95	0.639	0.779
37	333.84	334.77	333.30	0.821	0.922
38	343.25	345.19	344.66	0.654	0.809
39	354.71	354.61	359.51	0.982	0.449
40	369.52	362.63	379.64	0.420	0.285

Supplementary Table S7A. Back-transformed and pairwise comparison of weekly fetal growth biometrics across sum of even-chain SFAs tertiles in the NICHD Fetal Growth Studies-Singletons

GW	Estimated fetal weight, g					Abdominal circumference, mm					Femur length, mm				
	Back-transformed geometric mean gram			Wald test for pairwise comparison p value		Back-transformed geometric mean mm			Wald test for pairwise comparison p value		Back-transformed geometric mean			Wald test for pairwise comparison p value	
	1st tertile	2nd tertile	3rd tertile	2nd vs 1st tertile	3rd vs 1st tertile	1st tertile	2nd tertile	3rd tertile	2nd vs 1st tertile	3rd vs 1st tertile	1st tertile	2nd tertile	3rd tertile	2nd vs 1st tertile	3rd vs 1st tertile
10	31.44	41.46	35.96	**0.002**	0.073	34.40	44.93	36.03	**<0.0001**	0.307	1.67	2.97	2.66	**<0.0001**	**<0.0001**
11	41.77	48.67	43.96	**0.001**	0.218	44.53	51.78	45.66	**<0.0001**	0.328	3.45	4.73	4.31	**<0.0001**	**<0.0001**
12	54.94	58.75	54.56	**0.001**	0.769	55.79	59.75	56.19	**<0.0001**	0.632	6.14	7.02	6.48	**<0.0001**	**0.028**
13	71.53	72.48	68.47	0.452	**0.030**	67.85	68.9	67.36	0.130	0.578	9.63	9.78	9.12	0.295	**0.009**
14	92.19	90.82	86.53	0.478	**0.005**	80.39	79.23	78.92	0.235	0.198	13.53	12.89	12.11	**0.010**	**<0.0001**
15	117.58	114.87	109.67	0.287	**0.003**	93.07	90.67	90.64	**0.040**	0.073	17.42	16.19	15.31	**<0.001**	**<0.0001**
16	148.38	145.77	138.82	0.376	**0.003**	105.64	103.06	102.35	**0.034**	**0.027**	20.93	19.54	18.59	**<0.001**	**<0.0001**
17	185.24	184.44	174.77	0.812	**0.006**	117.94	116.12	114.00	0.139	**0.013**	23.93	22.84	21.86	**0.004**	**<0.0001**
18	228.72	231.26	217.96	0.537	**0.022**	129.92	129.42	125.62	0.707	**0.012**	26.56	26.05	25.10	0.221	**0.005**
19	279.30	285.9	268.51	0.208	0.065	141.65	142.48	137.26	0.588	**0.020**	29.15	29.21	28.33	0.905	0.155
20	337.50	348.43	326.93	0.088	0.138	153.10	155.06	148.85	0.238	**0.038**	31.82	32.27	31.47	0.401	0.581
21	403.91	419.14	393.77	**0.041**	0.233	164.22	167.06	160.32	0.101	0.075	34.56	35.18	34.49	0.266	0.921
22	479.14	498.28	469.61	**0.023**	0.342	174.99	178.42	171.64	0.052	0.149	37.34	37.93	37.35	0.306	0.986
23	563.85	586.14	555.07	**0.020**	0.459	185.44	189.14	182.79	**0.040**	0.280	40.12	40.49	40.02	0.541	0.893
24	658.79	683.08	650.86	**0.030**	0.573	195.65	199.31	193.78	0.053	0.476	42.88	42.87	42.51	0.978	0.637
25	764.86	789.65	757.81	0.064	0.675	205.71	209.05	204.64	0.104	0.705	45.59	45.10	44.83	0.472	0.374
26	883.12	906.59	876.94	0.141	0.757	215.80	218.55	215.46	0.217	0.911	48.20	47.21	47.01	0.186	0.196
27	1014.91	1035.03	1009.55	0.274	0.818	226.09	228.04	226.32	0.408	0.944	50.69	49.27	49.12	0.073	0.105
28	1161.88	1176.48	1157.30	0.465	0.863	236.84	237.81	237.36	0.679	0.876	53.00	51.33	51.21	**0.035**	0.069
29	1325.66	1332.84	1321.76	0.729	0.896	248.23	248.15	248.68	0.970	0.897	55.12	53.47	53.34	**0.029**	0.070
30	1506.06	1505.00	1502.34	0.962	0.912	260.15	259.07	260.18	0.625	0.994	57.07	55.69	55.51	0.060	0.118
31	1701.57	1693.18	1696.84	0.751	0.904	272.3	270.46	271.66	0.446	0.869	58.88	57.93	57.71	0.229	0.262
32	1909.43	1897.20	1901.60	0.707	0.865	284.33	282.17	282.92	0.436	0.736	60.63	60.14	59.90	0.588	0.521
33	2125.50	2116.46	2111.29	0.811	0.786	295.85	294.03	293.71	0.549	0.632	62.37	62.26	62.07	0.916	0.803
34	2344.21	2349.80	2318.86	0.888	0.659	306.39	305.84	303.77	0.856	0.570	64.18	64.24	64.18	0.949	0.998
35	2562.92	2595.01	2516.84	0.446	0.469	315.69	317.37	312.87	0.568	0.554	66.11	66.00	66.20	0.905	0.941
36	2784.87	2844.17	2709.98	0.255	0.305	324.45	328.39	321.5	0.253	0.568	68.09	67.55	68.04	0.620	0.972
37	3015.66	3083.73	2914.06	0.291	0.219	333.70	338.62	330.64	0.230	0.585	69.99	68.94	69.55	0.418	0.773
38	3263.23	3296.78	3150.31	0.630	0.211	344.59	347.80	341.36	0.447	0.580	71.68	70.25	70.59	0.281	0.481
39	3538.17	3464.08	3446.96	0.393	0.418	358.44	355.64	354.93	0.570	0.604	73.02	71.55	71.01	0.352	0.275
40	3854.39	3565.81	3842.83	0.109	0.953	376.82	361.85	372.80	0.144	0.724	73.85	72.90	70.66	0.772	0.345

Supplementary Table S7B. Back-transformed and pairwise comparison of weekly fetal growth biometrics across sum of even-chain saturated fatty acids tertiles in the NICHD Fetal Growth Studies-Singletons

GW	Head circumference, mm					Biparietal diameter, mm					HC/AC ratio				
	Back-transformed geometric mean mm			Wald test for pairwise comparison p value		Back-transformed geometric mean mm			Wald test for pairwise comparison p value		Back-transformed geometric mean			Wald test for pairwise comparison p value	
	1st tertile	2nd tertile	3rd tertile	2nd vs 1st tertile	3rd vs 1st tertile	1st tertile	2nd tertile	3rd tertile	2nd vs 1st tertile	3rd vs 1st tertile	1st tertile	2nd tertile	3rd tertile	2nd vs 1st tertile	3rd vs 1st tertile
10	47.46	55.48	46.80	**<0.001**	0.692	11.84	13.43	11.90	**0.007**	0.908	1.38	1.24	1.26	**0.007**	**0.024**
11	59.35	64.72	58.32	**<0.0001**	0.383	15.32	16.58	15.26	**0.001**	0.867	1.33	1.25	1.26	**0.002**	**0.007**
12	72.05	74.98	70.68	**<0.0001**	0.111	19.05	19.97	18.88	**<0.0001**	0.431	1.29	1.26	1.25	**0.003**	**0.003**
13	85.18	86.21	83.55	0.150	0.071	22.86	23.54	22.62	**0.002**	0.232	1.26	1.25	1.24	0.663	0.225
14	98.40	98.34	96.62	0.949	0.117	26.63	27.20	26.37	0.059	0.342	1.22	1.24	1.23	0.160	0.409
15	111.44	111.22	109.65	0.853	0.176	30.24	30.89	30.02	0.072	0.504	1.2	1.23	1.22	**0.017**	**0.037**
16	124.12	124.66	122.48	0.653	0.246	33.65	34.53	33.54	**0.016**	0.738	1.18	1.21	1.21	**0.002**	**0.003**
17	136.43	138.4	135.06	0.098	0.357	36.87	38.11	36.91	**0.001**	0.915	1.16	1.19	1.2	**<0.001**	**<0.0001**
18	148.47	152.13	147.46	**0.004**	0.530	39.96	41.59	40.18	**<0.0001**	0.560	1.14	1.18	1.18	**<0.001**	**<0.0001**
19	160.44	165.5	159.77	**<0.001**	0.702	43.04	44.99	43.41	**<0.0001**	0.368	1.13	1.16	1.17	**0.002**	**<0.0001**
20	172.35	178.35	171.90	**0.0001**	0.811	46.13	48.28	46.58	**<0.0001**	0.305	1.13	1.15	1.16	**0.010**	**0.0001**
21	184.13	190.58	183.78	**<0.0001**	0.859	49.21	51.45	49.66	**<0.0001**	0.314	1.12	1.14	1.15	**0.033**	**0.001**
22	195.71	202.15	195.34	**<0.0001**	0.859	52.26	54.48	52.66	**<0.0001**	0.393	1.12	1.13	1.14	0.097	**0.008**
23	207.04	213.06	206.55	**<0.001**	0.825	55.29	57.39	55.57	**<0.0001**	0.562	1.12	1.13	1.13	0.249	0.070
24	218.08	223.37	217.41	**0.003**	0.775	58.27	60.18	58.39	**<0.001**	0.818	1.12	1.12	1.12	0.507	0.389
25	228.81	233.17	227.92	**0.021**	0.724	61.20	62.88	61.13	**0.003**	0.903	1.11	1.12	1.11	0.768	0.982
26	239.23	242.63	238.13	0.097	0.684	64.08	65.51	63.81	**0.020**	0.668	1.11	1.11	1.1	0.908	0.554
27	249.34	251.91	248.10	0.230	0.663	66.9	68.13	66.46	0.058	0.499	1.1	1.1	1.09	0.876	0.333
28	259.16	261.25	257.91	0.326	0.670	69.68	70.77	69.10	0.087	0.390	1.09	1.1	1.09	0.632	0.273
29	268.72	270.85	267.62	0.288	0.716	72.39	73.49	71.76	0.071	0.338	1.08	1.09	1.08	0.237	0.372
30	277.91	280.57	277.13	0.175	0.803	75.03	76.24	74.41	**0.042**	0.356	1.07	1.08	1.07	**0.035**	0.757
31	286.60	290.11	286.25	0.099	0.916	77.54	78.93	76.99	**0.031**	0.448	1.05	1.07	1.06	**0.006**	0.653
32	294.65	299.14	294.79	0.063	0.968	79.88	81.48	79.45	**0.029**	0.583	1.04	1.06	1.04	**0.003**	0.282
33	301.92	307.27	302.54	**0.039**	0.867	82.02	83.78	81.71	**0.024**	0.717	1.02	1.04	1.03	**0.003**	0.127
34	308.26	314.10	309.28	**0.020**	0.786	83.90	85.72	83.72	**0.017**	0.832	1.01	1.03	1.02	**0.007**	0.071
35	313.67	319.28	314.84	**0.022**	0.763	85.51	87.21	85.41	**0.023**	0.903	0.99	1.01	1.01	0.089	0.083
36	318.40	323.00	319.35	0.102	0.820	86.94	88.30	86.90	0.114	0.965	0.98	0.98	0.99	0.701	0.175
37	322.77	325.81	323.14	0.352	0.933	88.26	89.19	88.40	0.351	0.895	0.97	0.96	0.98	0.712	0.231
38	327.11	328.30	326.61	0.720	0.912	89.57	90.08	90.16	0.617	0.592	0.95	0.94	0.96	0.698	0.136
39	331.78	331.08	330.12	0.860	0.751	90.97	91.17	92.43	0.874	0.291	0.92	0.93	0.94	0.563	0.083
40	337.15	334.79	334.11	0.764	0.714	92.56	92.66	95.49	0.969	0.269	0.9	0.93	0.93	0.164	0.167

HC/AC ratio, head circumference/abdominal circumference ratio.

Supplementary Table S8A. Back-transformed and pairwise comparison of weekly fetal growth biometrics across Heptadecanoic acid (17:0) tertiles in the NICHD Fetal Growth Studies-Singletons

GW	Estimated fetal weight, g					Abdominal circumference, mm					Femur length, mm				
	Back-transformed geometric mean gram			Wald test for pairwise comparison p value		Back-transformed geometric mean mm			Wald test for pairwise comparison p value		Back-transformed geometric mean mm			Wald test for pairwise comparison p value	
	1st tertile	2nd tertile	3rd tertile	2nd vs 1st tertile	3rd vs 1st tertile	1st tertile	2nd tertile	3rd tertile	2nd vs 1st tertile	3rd vs 1st tertile	1st tertile	2nd tertile	3rd tertile	2nd vs 1st tertile	3rd vs 1st tertile
10	32.59	34.34	37.61	0.382	0.103	38.57	35.61	40.28	**0.006**	0.390	2.25	2.38	2.91	0.302	**0.001**
11	42.65	43.47	45.73	0.572	0.146	47.75	45.41	48.52	**0.006**	0.569	4.07	4.07	4.55	0.982	**0.010**
12	55.36	55.24	56.58	0.918	0.379	57.77	56.21	57.77	**0.045**	0.998	6.54	6.38	6.69	0.286	0.379
13	71.25	70.33	70.92	0.483	0.815	68.45	67.71	68.01	0.411	0.601	9.54	9.25	9.30	0.166	0.212
14	90.93	89.50	89.66	0.434	0.544	79.60	79.63	79.14	0.978	0.678	12.8	12.52	12.29	0.344	0.070
15	115.06	113.61	113.85	0.547	0.669	91.07	91.7	91.06	0.614	0.994	16.05	16.00	15.54	0.896	0.162
16	144.33	143.51	144.53	0.789	0.955	102.71	103.71	103.57	0.478	0.553	19.12	19.48	18.93	0.397	0.644
17	179.47	180.03	182.66	0.884	0.454	114.46	115.53	116.45	0.492	0.190	21.99	22.83	22.34	0.082	0.438
18	221.18	223.78	228.8	0.604	0.153	126.3	127.15	129.44	0.624	0.057	24.80	26.00	25.70	**0.027**	0.068
19	270.2	275.06	282.85	0.448	0.062	138.21	138.65	142.23	0.818	**0.028**	27.67	29.05	29.00	**0.022**	**0.016**
20	327.34	334.24	344.72	0.387	**0.041**	150.09	150.03	154.61	0.977	**0.024**	30.59	32.05	32.21	**0.026**	**0.008**
21	393.41	401.81	414.71	0.388	**0.041**	161.88	161.25	166.50	0.780	**0.032**	33.51	34.96	35.27	**0.041**	**0.007**
22	469.24	478.23	493.20	0.445	0.058	173.51	172.25	177.86	0.605	0.058	36.41	37.75	38.15	0.076	**0.011**
23	555.66	563.93	580.65	0.562	0.101	184.96	183.05	188.71	0.464	0.123	39.25	40.39	40.83	0.153	**0.029**
24	653.54	659.33	677.70	0.738	0.189	196.23	193.65	199.14	0.361	0.261	42.02	42.90	43.31	0.299	0.094
25	763.76	764.89	785.25	0.957	0.333	207.32	204.12	209.28	0.292	0.482	44.69	45.28	45.61	0.515	0.266
26	887.22	881.11	904.59	0.810	0.515	218.30	214.55	219.34	0.250	0.730	47.25	47.56	47.77	0.753	0.560
27	1024.86	1008.58	1037.48	0.590	0.690	229.24	225.04	229.54	0.228	0.925	49.70	49.76	49.84	0.950	0.877
28	1177.69	1148.06	1186.36	0.401	0.814	240.24	235.74	240.19	0.223	0.987	52.03	51.95	51.91	0.940	0.898
29	1346.25	1300.4	1354.01	0.260	0.856	251.36	246.79	251.56	0.242	0.953	54.25	54.16	54.02	0.940	0.819
30	1529.87	1466.2	1540.62	0.177	0.828	262.51	258.13	263.57	0.289	0.768	56.36	56.39	56.19	0.979	0.863
31	1727.00	1645.99	1744.17	0.141	0.766	273.59	269.6	275.92	0.365	0.545	58.39	58.58	58.36	0.878	0.978
32	1935.12	1840.28	1960.83	0.138	0.703	284.48	281.03	288.25	0.464	0.365	60.37	60.66	60.51	0.818	0.904
33	2150.69	2049.66	2184.69	0.169	0.662	295.04	292.23	300.14	0.573	0.253	62.30	62.56	62.56	0.849	0.836
34	2369.05	2274.72	2407.58	0.254	0.659	305.13	302.96	311.13	0.678	0.196	64.24	64.20	64.47	0.979	0.856
35	2586.22	2514.06	2620.21	0.439	0.725	314.66	313.07	320.73	0.772	0.201	66.17	65.52	66.18	0.654	0.995
36	2802.81	2756.41	2823.32	0.664	0.850	323.72	322.97	329.24	0.899	0.275	67.97	66.70	67.70	0.416	0.849
37	3021.47	2983.93	3026.75	0.756	0.966	332.42	333.37	337.65	0.878	0.343	69.44	68.09	69.11	0.420	0.833
38	3246.42	3174.46	3244.36	0.581	0.988	340.93	345.05	347.12	0.524	0.287	70.38	70.06	70.50	0.851	0.945
39	3483.48	3303.28	3494.27	0.216	0.943	349.41	358.93	358.93	0.169	0.129	70.61	73.05	71.97	0.186	0.440
40	3740.33	3346.37	3800.12	0.060	0.765	358.06	376.06	374.55	0.039	0.069	69.92	77.58	73.62	**0.005**	0.153

Supplementary Table S8B. Back-transformed and pairwise comparison of weekly fetal growth biometrics across Heptadecanoic acid (17:0) tertiles in the NICHD Fetal Growth Studies-Singletons

GW	Head circumference, mm					Biparietal diameter, mm					HC/AC ratio				
	Back-transformed geometric mean mm			Wald test for pairwise comparison p value		Back-transformed geometric mean mm			Wald test for pairwise comparison p value		Back-transformed geometric mean			Wald test for pairwise comparison p value	
	1st tertile	2nd tertile	3rd tertile	2nd vs 1st tertile	3rd vs 1st tertile	1st tertile	2nd tertile	3rd tertile	2nd vs 1st tertile	3rd vs 1st tertile	1st tertile	2nd tertile	3rd tertile	2nd vs 1st tertile	3rd vs 1st tertile
10	49.54	46.49	53.20	**0.020**	0.112	11.94	11.71	13.06	0.570	0.068	1.25	1.29	1.31	0.342	0.308
11	61.07	58.41	62.73	**0.005**	0.263	15.59	15.32	16.03	0.349	0.284	1.27	1.28	1.29	0.614	0.501
12	73.33	71.21	73.20	**0.004**	0.873	19.46	19.20	19.26	0.139	0.389	1.27	1.26	1.26	0.509	0.681
13	86.00	84.48	84.52	0.064	**0.041**	23.40	23.15	22.69	0.187	**0.001**	1.26	1.25	1.24	0.061	**0.030**
14	98.81	97.89	96.61	0.356	**0.027**	27.22	27.03	26.27	0.428	**0.001**	1.25	1.23	1.22	0.054	**0.015**
15	111.52	111.13	109.33	0.732	0.068	30.83	30.71	29.91	0.662	**0.009**	1.23	1.21	1.20	0.092	**0.015**
16	124.00	124.02	122.52	0.983	0.243	34.20	34.14	33.58	0.856	0.095	1.21	1.20	1.18	0.210	**0.016**
17	136.21	136.52	135.98	0.819	0.859	37.35	37.34	37.21	0.981	0.698	1.19	1.18	1.17	0.554	**0.027**
18	148.25	148.72	149.51	0.752	0.357	40.41	40.40	40.77	0.989	0.356	1.17	1.17	1.15	0.949	0.077
19	160.15	160.82	162.86	0.686	0.073	43.45	43.45	44.26	0.984	0.061	1.16	1.16	1.14	0.561	0.214
20	171.85	172.83	175.87	0.584	**0.015**	46.46	46.53	47.65	0.854	**0.011**	1.14	1.15	1.14	0.301	0.464
21	183.29	184.67	188.41	0.470	**0.003**	49.42	49.61	50.92	0.651	**0.002**	1.13	1.14	1.13	0.141	0.813
22	194.44	196.27	200.43	0.365	**0.001**	52.35	52.69	54.07	0.424	**0.001**	1.12	1.14	1.13	0.059	0.795
23	205.29	207.57	211.90	0.287	**<0.001**	55.22	55.73	57.08	0.247	**<0.001**	1.12	1.13	1.12	**0.028**	0.466
24	215.86	218.55	222.82	0.242	**0.001**	58.04	58.73	59.97	0.152	**<0.001**	1.11	1.13	1.12	**0.021**	0.274
25	226.18	229.17	233.23	0.229	**0.001**	60.83	61.66	62.76	0.115	**0.001**	1.10	1.12	1.11	**0.025**	0.196
26	236.34	239.43	243.24	0.247	**0.003**	63.60	64.51	65.46	0.110	**0.004**	1.10	1.12	1.11	**0.034**	0.183
27	246.41	249.34	252.95	0.303	**0.009**	66.35	67.27	68.13	0.131	**0.009**	1.09	1.11	1.10	**0.045**	0.214
28	256.53	258.94	262.52	0.419	**0.019**	69.12	69.92	70.80	0.195	**0.016**	1.09	1.10	1.09	0.052	0.310
29	266.71	268.25	272.09	0.619	**0.034**	71.91	72.47	73.51	0.366	**0.019**	1.08	1.09	1.08	0.060	0.582
30	276.73	277.23	281.54	0.879	0.063	74.67	74.90	76.24	0.715	**0.023**	1.07	1.08	1.07	0.103	0.854
31	286.32	285.79	290.65	0.879	0.120	77.33	77.23	78.90	0.882	**0.035**	1.06	1.07	1.05	0.236	0.345
32	295.20	293.88	299.14	0.723	0.196	79.82	79.47	81.42	0.635	0.054	1.05	1.05	1.04	0.449	0.140
33	303.04	301.44	306.75	0.681	0.255	82.08	81.65	83.69	0.578	0.069	1.03	1.04	1.02	0.660	0.072
34	309.51	308.38	313.17	0.780	0.267	84.03	83.77	85.64	0.745	0.073	1.02	1.02	1.01	0.841	0.050
35	314.50	314.67	318.16	0.967	0.266	85.62	85.85	87.16	0.771	0.079	1.00	1.00	0.99	0.986	0.080
36	318.37	320.37	321.98	0.652	0.309	86.92	87.81	88.34	0.338	0.136	0.99	0.99	0.98	0.983	0.298
37	321.59	325.59	325.22	0.394	0.354	88.02	89.54	89.35	0.143	0.212	0.97	0.97	0.97	0.790	0.668
38	324.64	330.44	328.51	0.229	0.340	89.00	90.91	90.38	0.059	0.213	0.95	0.96	0.95	0.355	0.856
39	328.04	335.05	332.50	0.165	0.301	89.96	91.81	91.62	0.108	0.163	0.93	0.95	0.93	0.084	0.710
40	332.33	339.57	337.89	0.266	0.412	91.01	92.10	93.27	0.628	0.250	0.91	0.94	0.90	0.163	0.505

HC/AC ratio, head circumference/abdominal circumference ratio.

Supplementary Table S9A. Back-transformed and pairwise comparison of weekly fetal growth biometrics across sum of odd-chain saturated fatty acids tertiles in the NICHD Fetal Growth Studies-Singletons

GW	Estimated fetal weight, g					Femur length, mm				
	Back-transformed geometric mean gram			Wald test for pairwise comparison p value		Back-transformed geometric mean mm			Wald test for pairwise comparison p value	
	1st tertile	2nd tertile	3rd tertile	2nd vs 1st tertile	3rd vs 1st tertile	1st tertile	2nd tertile	3rd tertile	2nd vs 1st tertile	3rd vs 1st tertile
10	33.60	35.77	36.09	0.362	0.283	2.33	2.61	2.50	0.092	0.195
11	43.44	44.38	45.41	0.577	0.234	4.11	4.27	4.28	0.298	0.220
12	55.86	55.74	57.46	0.927	0.188	6.51	6.50	6.69	0.925	0.276
13	71.42	70.62	72.91	0.559	0.258	9.43	9.23	9.65	0.313	0.323
14	90.75	89.89	92.58	0.646	0.315	12.64	12.36	12.97	0.307	0.269
15	114.54	114.52	117.36	0.994	0.237	15.92	15.73	16.45	0.598	0.152
16	143.52	145.51	148.15	0.510	0.109	19.06	19.17	19.88	0.795	0.060
17	178.43	183.67	185.81	0.157	**0.032**	22.03	22.56	23.14	0.246	**0.022**
18	220.00	229.47	230.98	**0.042**	**0.009**	24.89	25.86	26.23	0.064	**0.013**
19	268.98	282.86	283.92	**0.016**	**0.004**	27.8	29.08	29.25	**0.030**	**0.015**
20	326.17	344.02	344.94	**0.011**	**0.004**	30.75	32.22	32.25	**0.022**	**0.022**
21	392.40	413.33	414.55	**0.011**	**0.004**	33.70	35.24	35.18	**0.025**	**0.036**
22	468.49	491.12	493.32	**0.019**	**0.007**	36.62	38.10	38.02	**0.040**	0.065
23	555.25	577.82	581.86	**0.045**	**0.013**	39.48	40.78	40.73	0.087	0.121
24	653.44	673.92	680.86	0.127	**0.031**	42.26	43.28	43.32	0.209	0.218
25	763.80	780.08	791.15	0.312	0.070	44.93	45.61	45.77	0.443	0.363
26	887.04	897.21	913.79	0.596	0.135	47.49	47.79	48.1	0.752	0.539
27	1023.79	1026.55	1050.10	0.902	0.208	49.93	49.88	50.31	0.960	0.714
28	1174.68	1169.79	1201.78	0.847	0.257	52.25	51.92	52.45	0.757	0.856
29	1340.05	1328.58	1370.81	0.683	0.252	54.45	53.97	54.54	0.657	0.942
30	1519.34	1503.5	1556.93	0.615	0.216	56.55	56.05	56.58	0.648	0.981
31	1711.13	1694.69	1757.36	0.651	0.188	58.56	58.13	58.57	0.714	0.997
32	1913.27	1901.89	1967.45	0.788	0.184	60.50	60.22	60.48	0.827	0.985
33	2122.78	2124.33	2180.45	0.974	0.215	62.39	62.31	62.28	0.955	0.942
34	2335.80	2360.66	2387.49	0.639	0.311	64.26	64.39	63.97	0.923	0.852
35	2548.11	2607.75	2579.39	0.304	0.572	66.11	66.45	65.53	0.814	0.714
36	2756.66	2856.28	2763.50	0.135	0.913	67.83	68.41	67.01	0.712	0.626
37	2958.92	3092.69	2961.71	0.082	0.969	69.24	70.15	68.51	0.598	0.689
38	3152.56	3300.40	3203.03	0.073	0.513	70.12	71.52	70.16	0.436	0.984
39	3335.59	3460.86	3526.18	0.164	**0.044**	70.28	72.38	72.06	0.264	0.376
40	3506.38	3555.38	3986.26	0.720	**0.005**	69.56	72.60	74.37	0.258	0.055

Supplementary Table S9B. Back-transformed and pairwise comparison of weekly fetal growth biometrics across sum of odd-chain saturated fatty acids tertiles in the NICHD Fetal Growth Studies-Singletons

GW	Head circumference, mm					Biparietal diameter, mm				
	Back-transformed geometric mean mm			Wald test for pairwise comparison p value		Back-transformed geometric mean mm			Wald test for pairwise comparison p value	
	1st tertile	2nd tertile	3rd tertile	2nd vs 1st tertile	3rd vs 1st tertile	1st tertile	2nd tertile	3rd tertile	2nd vs 1st tertile	3rd vs 1st tertile
10	50.81	47.07	51.48	**0.042**	0.695	12.14	11.71	12.87	0.410	0.178759
11	61.85	58.83	62.33	**0.013**	0.661	15.66	15.22	16.10	0.223	0.217932
12	73.67	71.52	74.00	**0.005**	0.611	19.39	19.03	19.57	0.094	0.378239
13	86.01	84.79	86.28	0.099	0.675	23.18	22.97	23.19	0.333	0.937566
14	98.65	98.32	98.96	0.730	0.716	26.88	26.91	26.86	0.933	0.941149
15	111.38	111.83	111.84	0.688	0.639	30.42	30.72	30.50	0.365	0.787813
16	124.03	125.12	124.76	0.364	0.483	33.74	34.35	34.06	0.088	0.348725
17	136.52	138.1	137.59	0.215	0.315	36.90	37.77	37.49	**0.018**	0.079
18	148.81	150.77	150.28	0.162	0.206	39.98	41.05	40.83	**0.009**	**0.022**
19	160.89	163.28	162.82	0.121	0.130	43.06	44.28	44.09	**0.07**	**0.011**
20	172.67	175.60	175.18	0.072	0.067	46.11	47.48	47.30	**0.004**	**0.006**
21	184.11	187.67	187.27	**0.033**	**0.026**	49.12	50.63	50.45	**0.002**	**0.003**
22	195.17	199.40	199.04	**0.012**	**0.008**	52.09	53.73	53.52	**0.001**	**0.001**
23	205.86	210.75	210.43	**0.005**	**0.002**	55.00	56.76	56.51	**<0.001**	**<0.001**
24	216.24	221.70	221.45	**0.003**	**0.001**	57.84	59.71	59.41	**<0.001**	**0.001**
25	226.37	232.24	232.08	**0.003**	**0.001**	60.62	62.57	62.24	**<0.001**	**0.002**
26	236.37	242.39	242.38	**0.006**	**0.001**	63.34	65.35	65.01	**0.001**	**0.003**
27	246.37	252.20	252.39	**0.011**	**0.002**	66.01	68.05	67.75	**0.002**	**0.004**
28	256.53	261.72	262.19	**0.023**	**0.003**	68.64	70.68	70.47	**0.002**	**0.002**
29	266.91	271.02	271.88	0.063	**0.008**	71.24	73.25	73.20	**<0.001**	**<0.001**
30	277.23	280.04	281.37	0.200	**0.027**	73.77	75.76	75.93	**<0.001**	**<0.0001**
31	287.14	288.70	290.41	0.504	0.102	76.22	78.18	78.58	**0.002**	**<0.0001**
32	296.23	296.93	298.71	0.786	0.260	78.55	80.53	81.06	**0.006**	**<0.001**
33	304.08	304.65	305.96	0.833	0.419	80.73	82.79	83.26	**0.007**	**<0.001**
34	310.23	311.79	311.86	0.556	0.477	82.72	84.95	85.10	**0.003**	**<0.001**
35	314.46	318.26	316.14	0.143	0.452	84.48	87.00	86.48	**0.001**	**<0.001**
36	317.33	323.94	319.28	**0.022**	0.428	85.91	88.91	87.54	**<0.001**	**0.037**
37	319.57	328.73	322.17	**0.005**	0.343	86.91	90.6	88.57	**<0.001**	0.067
38	321.96	332.50	325.79	**0.001**	0.164	87.37	92.03	89.85	**<0.0001**	**0.006**
39	325.27	335.14	331.11	**0.004**	0.073	87.20	93.13	91.69	**<0.0001**	**<0.0001**
40	330.34	336.56	339.23	0.314	0.170	86.31	93.84	94.45	**<0.0001**	**<0.001**

Supplementary Table S10A. Back-transformed and pairwise comparison of weekly fetal growth biometrics across lignoceric acid (24:0) tertiles in the NICHD Fetal Growth Studies-Singletons

GW	Estimated fetal weight (EFW), g					Abdominal circumference (AC), mm					Femur length (FL), mm				
	Back-transformed geometric mean gram			Wald test for pairwise comparison p value		Back-transformed geometric mean mm			Wald test for pairwise comparison p value		Back-transformed geometric mean			Wald test for pairwise comparison p value	
	1st tertile	2nd tertile	3rd tertile	2nd vs 1st tertile	3rd vs 1st tertile	1st tertile	2nd tertile	3rd tertile	2nd vs 1st tertile	3rd vs 1st tertile	1st tertile	2nd tertile	3rd tertile	2nd vs 1st tertile	3rd vs 1st tertile
10	37.47	35.31	37.65	0.246	0.945	41.76	35.89	40.33	**<0.001**	0.494	2.81	2.06	2.85	**<0.0001**	0.859
11	45.81	43.84	45.92	0.122	0.945	49.72	45.85	48.28	**<0.001**	0.263	4.49	3.82	4.56	**<0.0001**	0.710
12	56.75	55.10	56.85	0.061	0.919	58.61	56.81	57.24	**0.013**	0.056	6.71	6.31	6.80	**<0.001**	0.464
13	70.98	69.84	71.16	0.225	0.879	68.42	68.46	67.21	0.961	0.089	9.40	9.44	9.51	0.764	0.471
14	89.32	88.92	89.68	0.767	0.832	79.07	80.50	78.12	0.145	0.333	12.45	12.98	12.55	**0.025**	0.673
15	112.67	113.29	113.37	0.729	0.756	90.46	92.65	89.85	0.060	0.594	15.73	16.62	15.80	**0.004**	0.830
16	141.98	143.91	143.19	0.391	0.654	102.44	104.7	102.21	0.074	0.847	19.08	20.11	19.10	**0.004**	0.968
17	178.08	181.53	179.98	0.211	0.557	114.81	116.52	114.95	0.200	0.910	22.40	23.33	22.36	**0.019**	0.913
18	221.54	226.55	224.27	0.148	0.503	127.33	128.13	127.78	0.583	0.750	25.65	26.31	25.58	0.134	0.861
19	272.50	278.81	276.22	0.150	0.472	139.76	139.6	140.41	0.918	0.678	28.85	29.23	28.76	0.447	0.847
20	331.36	338.39	336.30	0.198	0.437	151.96	150.91	152.71	0.548	0.657	31.97	32.11	31.84	0.801	0.812
21	398.71	405.60	405.03	0.302	0.404	163.85	162.00	164.57	0.325	0.683	34.97	34.93	34.80	0.940	0.757
22	475.18	480.79	482.92	0.486	0.384	175.40	172.86	175.93	0.202	0.767	37.82	37.66	37.58	0.795	0.690
23	561.43	564.39	570.41	0.760	0.392	186.61	183.47	186.79	0.138	0.923	40.49	40.28	40.19	0.742	0.627
24	658.23	657.01	667.96	0.917	0.439	197.54	193.88	197.19	0.106	0.858	42.99	42.78	42.63	0.760	0.584
25	766.52	759.51	776.04	0.619	0.532	208.29	204.16	207.23	0.090	0.620	45.33	45.17	44.93	0.821	0.571
26	887.43	873.11	895.16	0.397	0.673	219.00	214.41	217.06	0.079	0.403	47.55	47.45	47.12	0.895	0.579
27	1022.41	999.48	1025.94	0.253	0.870	229.88	224.75	226.85	0.067	0.218	49.70	49.65	49.27	0.952	0.604
28	1173.27	1140.89	1169.13	0.168	0.866	241.15	235.37	236.85	**0.049**	0.081	51.83	51.79	51.45	0.965	0.644
29	1341.96	1299.72	1325.47	0.120	0.546	253.04	246.40	247.25	**0.029**	**0.014**	54.01	53.92	53.71	0.909	0.703
30	1528.28	1475.76	1494.93	0.096	0.280	265.45	257.76	257.99	**0.016**	**0.001**	56.25	56.02	56.03	0.787	0.773
31	1730.03	1666.98	1676.94	0.088	0.141	278.09	269.29	268.9	**0.010**	**<0.001**	58.50	58.07	58.32	0.637	0.834
32	1943.40	1869.74	1870.54	0.088	0.089	290.62	280.79	279.81	**0.007**	**<0.001**	60.70	60.04	60.51	0.498	0.844
33	2162.70	2078.55	2074.31	0.090	0.073	302.65	292.07	290.51	**0.007**	**0.0001**	62.79	61.88	62.50	0.380	0.777
34	2380.24	2285.90	2286.35	0.091	0.084	313.72	302.85	300.74	**0.008**	**<0.0001**	64.70	63.57	64.18	0.277	0.606
35	2587.39	2485.65	2503.97	0.101	0.159	323.40	312.97	310.31	**0.014**	**<0.0001**	66.37	65.07	65.49	0.210	0.358
36	2784.15	2685.22	2722.53	0.159	0.363	331.92	322.81	319.38	**0.045**	**<0.001**	67.85	66.53	66.59	0.234	0.238
37	2978.31	2899.55	2936.06	0.318	0.591	340.17	333.04	328.28	0.145	**0.003**	69.29	68.13	67.73	0.340	0.217
38	3181.02	3148.79	3137.60	0.708	0.613	349.19	344.41	337.35	0.349	**0.005**	70.87	70.10	69.18	0.533	0.201
39	3406.85	3459.97	3319.40	0.595	0.355	360.09	357.79	347.01	0.689	**0.005**	72.78	72.69	71.25	0.952	0.291
40	3674.53	3870.51	3473.30	0.198	0.161	374.16	374.18	357.71	0.998	0.068	75.22	76.19	74.27	0.730	0.738

Supplementary Table S10B. Back-transformed and pairwise comparison of weekly fetal growth biometrics across lignoceric acid (24:0) tertiles in the NICHD Fetal Growth Studies-Singletons

GW	Head circumference (HC), mm					Biparietal diameter (BPD), mm					HC/AC ratio				
	Back-transformed geometric mean mm			Wald test for pairwise comparison p value		Back-transformed geometric mean mm			Wald test for pairwise comparison p value		Back-transformed geometric mean			Wald test for pairwise comparison p value	
	1st tertile	2nd tertile	3rd tertile	2nd vs 1st tertile	3rd vs 1st tertile	1st tertile	2nd tertile	3rd tertile	2nd vs 1st tertile	3rd vs 1st tertile	1st tertile	2nd tertile	3rd tertile	2nd vs 1st tertile	3rd vs 1st tertile
10	48.84	46.33	51.18	**0.043**	0.186	13.25	11.63	12.62	**0.002**	0.278	1.27	1.26	1.28	0.899	0.671
11	60.23	58.34	61.69	**0.044**	0.225	16.28	15.30	15.82	**0.005**	0.213	1.26	1.25	1.29	0.737	0.312
12	72.46	71.25	72.97	0.113	0.554	19.56	19.26	19.25	0.143	0.143	1.26	1.25	1.28	0.402	**0.035**
13	85.24	84.68	84.85	0.491	0.682	23.02	23.31	22.80	0.115	0.318	1.24	1.23	1.26	0.208	**0.038**
14	98.30	98.25	97.15	0.964	0.325	26.59	27.27	26.40	**0.006**	0.522	1.23	1.22	1.24	0.269	0.274
15	111.40	111.66	109.68	0.821	0.197	30.20	31.01	29.95	**0.006**	0.471	1.22	1.21	1.22	0.303	0.768
16	124.39	124.70	122.29	0.806	0.146	33.81	34.49	33.42	**0.029**	0.277	1.20	1.19	1.20	0.295	0.571
17	137.19	137.3	134.89	0.934	0.139	37.38	37.71	36.78	0.295	0.109	1.19	1.18	1.17	0.289	0.124
18	149.81	149.53	147.42	0.849	0.161	40.89	40.75	40.07	0.686	**0.044**	1.18	1.17	1.15	0.321	**0.020**
19	162.36	161.57	159.83	0.627	0.180	44.34	43.77	43.31	0.114	**0.021**	1.16	1.16	1.14	0.376	**0.005**
20	174.76	173.42	172.04	0.442	0.179	47.72	46.77	46.48	**0.014**	**0.010**	1.16	1.15	1.13	0.434	**0.002**
21	186.94	184.99	183.95	0.300	0.162	51.01	49.76	49.58	**0.002**	**0.004**	1.15	1.14	1.12	0.499	**<0.001**
22	198.81	196.25	195.51	0.200	0.141	54.18	52.72	52.58	**<0.001**	**0.001**	1.14	1.14	1.11	0.589	**<0.001**
23	210.34	207.15	206.68	0.136	0.122	57.24	55.63	55.48	**<0.0001**	**0.001**	1.13	1.13	1.11	0.727	**<0.001**
24	221.47	217.70	217.45	0.098	0.111	60.18	58.49	58.29	**<0.0001**	**<0.001**	1.13	1.13	1.10	0.917	**0.003**
25	232.22	227.90	227.84	0.078	0.108	63.04	61.3	61.02	**<0.001**	**<0.001**	1.12	1.12	1.10	0.865	**0.018**
26	242.57	237.80	237.90	0.068	0.109	65.81	64.06	63.68	**<0.001**	**<0.001**	1.11	1.12	1.10	0.646	0.087
27	252.58	247.46	247.69	0.065	0.113	68.54	66.75	66.30	**<0.001**	**<0.001**	1.10	1.11	1.09	0.430	0.286
28	262.28	256.96	257.31	0.068	0.118	71.27	69.4	68.91	**<0.001**	**<0.001**	1.09	1.10	1.09	0.223	0.683
29	271.74	266.35	266.83	0.077	0.130	74.02	72.01	71.53	**<0.0001**	**<0.0001**	1.08	1.09	1.08	0.071	0.727
30	280.85	275.52	276.13	0.096	0.158	76.76	74.54	74.13	**<0.0001**	**<0.0001**	1.06	1.08	1.07	**0.017**	0.237
31	289.47	284.29	285.02	0.125	0.209	79.43	76.97	76.65	**<0.0001**	**<0.0001**	1.05	1.06	1.06	**0.008**	0.069
32	297.47	292.47	293.32	0.162	0.275	81.96	79.28	79.04	**<0.0001**	**<0.0001**	1.03	1.05	1.05	**0.009**	**0.033**
33	304.69	299.85	300.81	0.198	0.334	84.29	81.43	81.22	**<0.0001**	**<0.0001**	1.02	1.03	1.03	**0.013**	**0.022**
34	310.97	306.23	307.28	0.223	0.368	86.33	83.39	83.13	**<0.0001**	**<0.0001**	1.00	1.02	1.02	**0.023**	**0.018**
35	316.18	311.48	312.55	0.245	0.382	88.00	85.13	84.71	**<0.0001**	**<0.0001**	0.99	1.00	1.01	0.103	**0.029**
36	320.43	315.93	316.59	0.293	0.384	89.34	86.70	85.93	**<0.001**	**<0.0001**	0.98	0.98	0.99	0.542	0.178
37	324.02	320.05	319.41	0.377	0.333	90.46	88.12	86.77	**0.005**	**<0.001**	0.97	0.97	0.97	0.879	0.682
38	327.30	324.32	321.06	0.515	0.202	91.47	89.44	87.25	**0.011**	**<0.0001**	0.95	0.95	0.95	0.490	0.645
39	330.63	329.25	321.60	0.778	0.071	92.51	90.73	87.35	0.067	**<0.0001**	0.94	0.93	0.93	0.490	0.178
40	334.38	335.39	321.09	0.880	**0.046**	93.70	92.02	87.08	0.449	**<0.001**	0.92	0.92	0.90	0.885	0.231

HC/AC ratio, head circumference/abdominal circumference ratio.

Supplementary Table S11. Back-transformed and pairwise comparison of weekly fetal growth biometrics across arachidic acid (20:0) tertiles in the NICHD Fetal Growth Studies-Singletons

GW	Femur length, mm					Head circumference, mm					Biparietal diameter, mm				
	Back-transformed geometric mean mm			Wald test for pairwise comparison p value		Back-transformed geometric mean gram			Wald test for pairwise comparison p value		Back-transformed geometric mean mm			Wald test for pairwise comparison p value	
	1st tertile	2nd tertile	3rd tertile	2nd vs 1st tertile	3rd vs 1st tertile	1st tertile	2nd tertile	3rd tertile	2nd vs 1st tertile	3rd vs 1st tertile	1st tertile	2nd tertile	3rd tertile	2nd vs 1st tertile	3rd vs 1st tertile
10	2.24	2.65	2.37	**0.006**	0.336	52.07	51.06	46.51	0.570	**0.003**	12.94	13.19	10.93	0.612	**<0.0001**
11	3.99	4.48	4.06	**0.001**	0.668	62.47	61.99	58.75	0.674	**0.003**	16.10	16.38	14.75	0.403	**0.0001**
12	6.43	6.90	6.33	**<0.0001**	0.565	73.69	73.7	71.85	0.986	**0.010**	19.49	19.77	18.88	0.160	**0.003**
13	9.46	9.83	9.13	**0.014**	0.138	85.56	85.97	85.39	0.539	0.779	23.02	23.29	23.10	0.170	0.650
14	12.88	13.07	12.29	0.431	0.063	97.91	98.57	98.99	0.455	0.183	26.60	26.85	27.21	0.354	**0.014**
15	16.43	16.40	15.63	0.943	0.056	110.56	111.31	112.34	0.472	0.067	30.16	30.37	31.04	0.507	**0.003**
16	19.86	19.67	18.97	0.609	0.079	123.37	124.05	125.28	0.536	0.062	33.66	33.82	34.55	0.621	**0.004**
17	23.06	22.77	22.22	0.518	0.161	136.21	136.68	137.79	0.666	0.136	37.06	37.18	37.78	0.722	**0.027**
18	26.03	25.75	25.41	0.603	0.377	148.98	149.21	150.02	0.844	0.373	40.39	40.47	40.84	0.807	0.197
19	28.89	28.70	28.58	0.765	0.706	161.63	161.64	162.15	0.992	0.688	43.68	43.74	43.9	0.862	0.565
20	31.74	31.63	31.71	0.878	0.974	174.07	173.9	174.14	0.904	0.962	46.93	46.98	46.96	0.903	0.943
21	34.55	34.49	34.75	0.943	0.857	186.22	185.92	185.9	0.833	0.828	50.12	50.16	49.99	0.934	0.766
22	37.29	37.26	37.64	0.972	0.770	198.01	197.6	197.38	0.784	0.673	53.25	53.27	52.99	0.954	0.560
23	39.95	39.91	40.38	0.976	0.744	209.4	208.92	208.53	0.762	0.569	56.29	56.31	55.93	0.964	0.426
24	42.50	42.44	42.96	0.963	0.758	220.35	219.84	219.33	0.761	0.516	59.25	59.27	58.81	0.964	0.350
25	44.95	44.84	45.37	0.939	0.799	230.89	230.36	229.76	0.772	0.505	62.13	62.16	61.61	0.954	0.309
26	47.30	47.13	47.64	0.912	0.853	241.04	240.49	239.84	0.783	0.515	64.93	64.98	64.32	0.933	0.278
27	49.57	49.32	49.80	0.883	0.908	250.86	250.28	249.61	0.782	0.524	67.66	67.74	66.95	0.900	0.230
28	51.78	51.44	51.91	0.858	0.954	260.43	259.79	259.11	0.759	0.513	70.35	70.47	69.49	0.851	0.156
29	53.95	53.53	54.01	0.838	0.981	269.82	269.05	268.43	0.704	0.481	73.01	73.18	71.93	0.790	0.074
30	56.08	55.59	56.13	0.825	0.984	278.93	278	277.52	0.644	0.473	75.61	75.82	74.29	0.732	**0.029**
31	58.16	57.61	58.26	0.824	0.969	287.59	286.52	286.25	0.623	0.519	78.10	78.37	76.57	0.687	**0.017**
32	60.16	59.61	60.37	0.837	0.942	295.63	294.52	294.48	0.647	0.613	80.43	80.78	78.78	0.645	**0.019**
33	62.06	61.57	62.42	0.864	0.909	302.86	301.89	302.02	0.710	0.733	82.54	82.99	80.93	0.584	**0.032**
34	63.86	63.51	64.40	0.907	0.874	309.08	308.53	308.73	0.831	0.886	84.39	84.95	83.03	0.485	0.075
35	65.54	65.42	66.25	0.969	0.842	314.21	314.33	314.41	0.960	0.933	85.93	86.63	85.09	0.376	0.278
36	67.08	67.28	67.94	0.953	0.819	318.63	319.33	318.94	0.801	0.912	87.25	88.02	86.98	0.374	0.763
37	68.47	69.07	69.42	0.870	0.814	322.93	323.62	322.2	0.824	0.819	88.47	89.14	88.48	0.490	0.986
38	69.70	70.76	70.62	0.787	0.827	327.68	327.32	324.07	0.909	0.253	89.72	90.03	89.36	0.756	0.717
39	70.76	72.30	71.49	0.713	0.869	333.54	330.55	324.46	0.425	**0.010**	91.13	90.72	89.37	0.722	0.107
40	71.64	73.67	71.98	0.679	0.946	341.18	333.45	323.31	0.276	**0.011**	92.86	91.26	88.32	0.454	0.031

Supplementary Table S12A. Back-transformed and pairwise comparison of weekly fetal growth biometrics across Behenic acid (22:0) tertiles in the NICHD Fetal Growth Studies-Singletons

GW	Estimated fetal weight, g					Abdominal circumference, mm				
	Back-transformed geometric mean gram			Wald test for pairwise comparison p value		Back-transformed geometric mean mm			Wald test for pairwise comparison p value	
	1st tertile	2nd tertile	3rd tertile	2nd vs 1st tertile	3rd vs 1st tertile	1st tertile	2nd tertile	3rd tertile	2nd vs 1st tertile	3rd vs 1st tertile
10	33.88	38.61	37.12	0.060	0.228	40.17	42.07	36.27	0.286	**0.033**
11	43.44	45.34	46.10	0.253	0.155	48.88	48.85	46.47	0.976	**0.046**
12	55.75	54.79	57.74	0.402	0.111	58.61	56.74	57.55	**0.004**	0.132
13	71.49	67.69	72.73	**0.004**	0.314	69.26	65.80	69.16	**<0.0001**	0.869
14	91.43	84.98	91.87	**0.001**	0.800	80.73	76.05	80.98	**<0.0001**	0.769
15	116.40	107.75	116.00	**<0.001**	0.860	92.86	87.42	92.75	**<0.0001**	0.920
16	147.26	137.10	145.99	**<0.001**	0.649	105.45	99.77	104.32	**<0.0001**	0.300
17	184.76	173.99	182.57	**0.002**	0.520	118.3	112.81	115.67	**<0.0001**	**0.021**
18	229.49	218.86	226.23	**0.015**	0.445	131.18	126.13	126.93	**<0.0001**	**0.001**
19	281.80	271.39	277.18	0.058	0.389	143.87	139.25	138.24	**0.001**	**<0.0001**
20	342.18	331.56	335.95	0.113	0.338	156.22	151.87	149.54	**0.005**	**<0.0001**
21	411.21	399.63	403.11	0.143	0.290	168.14	163.88	160.77	**0.008**	**<0.0001**
22	489.45	475.82	479.24	0.138	0.251	179.59	175.23	171.90	**0.008**	**<0.0001**
23	577.46	560.38	564.94	0.109	0.225	190.56	185.92	182.90	**0.006**	**<0.0001**
24	675.84	653.66	660.84	0.078	0.217	201.11	196.03	193.77	**0.004**	**<0.0001**
25	785.27	756.16	767.69	0.053	0.225	211.34	205.70	204.53	**0.004**	**<0.001**
26	906.54	868.65	886.35	**0.034**	0.242	221.40	215.14	215.24	**0.003**	**0.003**
27	1040.59	992.22	1017.87	**0.021**	0.261	231.46	224.59	225.97	**0.002**	**0.012**
28	1188.62	1128.43	1163.55	**0.011**	0.276	241.78	234.36	236.80	**0.001**	**0.023**
29	1351.68	1279.21	1324.85	**0.005**	0.293	252.51	244.77	247.84	**<0.001**	**0.026**
30	1529.40	1445.74	1501.74	**0.004**	0.331	263.57	255.82	259.05	**<0.001**	**0.029**
31	1720.47	1628.65	1692.48	**0.005**	0.393	274.79	267.39	270.27	**0.001**	**0.043**
32	1922.67	1828.41	1894.09	**0.016**	0.456	285.98	279.32	281.32	**0.009**	0.065
33	2132.82	2045.22	2102.21	0.053	0.488	296.93	291.42	292.01	0.051	0.073
34	2346.69	2278.99	2310.98	0.175	0.464	307.38	303.48	302.11	0.167478	0.055
35	2560.58	2528.79	2513.30	0.553	0.375	317.21	315.24	311.39	0.469981	**0.035**
36	2774.96	2787.92	2704.15	0.831	0.251	326.77	326.50	319.80	0.928129	**0.034**
37	2992.16	3043.80	2881.43	0.466	0.122	336.60	337.14	327.45	0.880475	**0.018**
38	3215.82	3280.04	3044.01	0.413	**0.024**	347.30	347.07	334.47	0.949557	**<0.001**
39	3451.05	3477.22	3191.62	0.766	**0.002**	359.55	356.16	341.04	0.452523	**<0.0001**
40	3704.55	3614.42	3324.84	0.517	**0.010**	374.12	364.32	347.32	0.305199	**0.009**

Supplementary Table S12B. Back-transformed and pairwise comparison of weekly fetal growth biometrics across Behenic acid (22:0) tertiles in the NICHD Fetal Growth Studies-Singletons

GW	HC, mm					BPD, mm				
	Back-transformed geometric mean mm			Wald test for pairwise comparison p value		Back-transformed geometric mean mm			Wald test for pairwise comparison p value	
	1st tertile	2nd tertile	3rd tertile	2nd vs 1st tertile	3rd vs 1st tertile	1st tertile	2nd tertile	3rd tertile	2nd vs 1st tertile	3rd vs 1st tertile
10	47.17	53.18	50.23	**<0.001**	0.075	11.46	13.26	12.17	**<0.0001**	0.121
11	59.39	62.42	61.67	**0.007**	0.057	15.13	16.16	15.75	**0.001**	0.066
12	72.41	72.60	73.90	0.811	0.068	19.08	19.31	19.58	0.298	**0.034**
13	85.82	83.67	86.64	**0.011**	0.297	23.11	22.65	23.49	0.057	0.100
14	99.23	95.55	99.62	**<0.001**	0.693	27.05	26.11	27.35	**0.003**	0.314
15	112.38	108.09	112.62	**<0.001**	0.832	30.77	29.64	31.03	**0.002**	0.449
16	125.08	121.14	125.47	**0.003**	0.755	34.23	33.19	34.51	**0.007**	0.450
17	137.36	134.48	138.11	**0.036**	0.574	37.44	36.71	37.79	0.071	0.384
18	149.37	147.86	150.55	0.313	0.416	40.52	40.19	40.94	0.444	0.327
19	161.35	161.01	162.87	0.838	0.341	43.60	43.60	44.08	0.990	0.310
20	173.29	173.77	175.00	0.786	0.320	46.70	46.92	47.20	0.673	0.326
21	185.11	186.03	186.85	0.623	0.335	49.81	50.14	50.28	0.541	0.371
22	196.75	197.73	198.37	0.614	0.387	52.89	53.23	53.31	0.549	0.453
23	208.14	208.84	209.51	0.730	0.483	55.95	56.20	56.27	0.676	0.585
24	219.23	219.38	220.26	0.947	0.621	58.97	59.05	59.15	0.905	0.767
25	230.00	229.40	230.61	0.794	0.780	61.94	61.79	61.96	0.822	0.978
26	240.43	239.01	240.6	0.566	0.943	64.84	64.44	64.67	0.579	0.814
27	250.51	248.33	250.28	0.405	0.926	67.67	67.03	67.31	0.406	0.626
28	260.26	257.53	259.72	0.311	0.835	70.43	69.61	69.86	0.303	0.467
29	269.66	266.75	268.99	0.285	0.799	73.10	72.20	72.35	0.265	0.341
30	278.64	275.93	278.04	0.329	0.825	75.66	74.79	74.76	0.293	0.265
31	287.08	284.88	286.72	0.451	0.898	78.08	77.34	77.09	0.395	0.245
32	294.89	293.39	294.86	0.633	0.991	80.33	79.79	79.34	0.563	0.274
33	301.96	301.23	302.29	0.828	0.920	82.39	82.09	81.49	0.766	0.352
34	308.19	308.18	308.82	0.997	0.847	84.22	84.18	83.55	0.972	0.501
35	313.55	313.98	314.29	0.900	0.828	85.81	86.00	85.50	0.850	0.763
36	318.26	318.67	318.49	0.907	0.950	87.20	87.53	87.22	0.759	0.992
37	322.57	322.51	321.24	0.988	0.738	88.46	88.80	88.46	0.771	0.999
38	326.76	325.78	322.39	0.807	0.274	89.65	89.84	89.00	0.876	0.594
39	331.14	328.79	321.79	0.582	**0.026**	90.84	90.68	88.6	0.907	0.081
40	336.02	331.85	319.31	0.512	**0.013**	92.08	91.36	87.05	0.711	**0.015**

Supplementary Table S13A. Back-transformed and pairwise comparison of weekly fetal growth biometrics across sum of very-long-even-chain saturated fatty acids tertiles in the NICHD Fetal Growth Studies-Singletons

GW	EFW, g					AC, mm				
	Back-transformed geometric mean gram			Wald test for pairwise comparison p value		Back-transformed geometric mean mm			Wald test for pairwise comparison p value	
	1st tertile	2nd tertile	3rd tertile	2nd vs 1st tertile	3rd vs 1st tertile	1st tertile	2nd tertile	3rd tertile	2nd vs 1st tertile	3rd vs 1st tertile
10	32.15	35.36	37.55	0.059	**0.027**	38.31	42.46	36.83	**0.028**	0.417
11	42.21	43.57	46.46	0.301	**0.013**	47.63	49.16	46.85	0.199	0.517
12	55.08	54.39	58.01	0.550	**0.012**	57.95	56.96	57.72	0.141	0.743
13	71.38	68.50	72.90	**0.038**	0.204	69.12	65.95	69.13	**<0.0001**	0.983
14	91.80	86.73	91.92	**0.007**	0.945	80.94	76.12	80.79	**<0.0001**	0.868
15	117.10	110.00	115.94	**0.003**	0.604	93.22	87.41	92.46	**<0.0001**	0.490
16	148.04	139.21	145.84	**0.004**	0.423	105.74	99.66	104.02	**<0.0001**	0.135
17	185.37	175.16	182.44	**0.006**	0.381	118.34	112.58	115.44	**<0.0001**	**0.016**
18	229.76	218.29	226.29	**0.014**	0.409	130.87	125.74	126.85	**<0.0001**	**0.002**
19	281.72	268.68	277.68	**0.026**	0.443	143.22	138.67	138.33	**0.001**	**0.001**
20	341.83	326.67	337.18	**0.036**	0.466	155.30	151.12	149.81	**0.006**	**<0.001**
21	410.73	392.74	405.41	**0.041**	0.480	167.01	162.97	161.23	**0.010**	**<0.001**
22	489.00	467.37	482.98	**0.041**	0.490	178.32	174.17	172.53	**0.010**	**<0.001**
23	577.23	551.07	570.45	**0.039**	0.505	189.22	184.75	183.68	**0.007**	**0.001**
24	676.03	644.44	668.43	**0.036**	0.528	199.75	194.77	194.68	**0.004**	**0.005**
25	786.01	748.21	777.52	**0.035**	0.556	210.01	204.38	205.55	**0.003**	**0.022**
26	907.86	863.30	898.38	**0.035**	0.582	220.10	213.79	216.31	**0.002**	0.075
27	1042.34	990.94	1031.75	**0.038**	0.599	230.19	223.23	227.03	**0.001**	0.159
28	1190.37	1132.68	1178.51	**0.047**	0.603	240.50	233.02	237.79	**<0.001**	0.230
29	1352.79	1290.15	1339.53	0.059	0.599	251.19	243.46	248.68	**<0.001**	0.245
30	1529.36	1463.47	1514.55	0.085	0.599	262.19	254.52	259.65	**<0.001**	0.232
31	1719.07	1651.74	1702.34	0.126	0.607	273.34	266.09	270.61	**0.001**	0.233
32	1920.25	1853.13	1900.87	0.185	0.613	284.46	278.00	281.43	**0.010**	0.244
33	2130.45	2064.81	2107.28	0.254	0.598	295.33	290.07	291.99	0.057	0.239
34	2346.44	2282.76	2317.78	0.324	0.554	305.71	302.08	302.15	0.191	0.211
35	2564.55	2502.46	2527.54	0.388	0.487	315.44	313.79	311.75	0.533	0.195
36	2782.18	2726.16	2730.22	0.485	0.404	324.92	325.05	320.68	0.964	0.207
37	2997.14	2962.55	2918.28	0.699	0.275	334.77	335.87	328.80	0.753	0.132
38	3207.39	3223.83	3083.48	0.868	0.104	345.71	346.24	336.02	0.884	**0.014**
39	3411.10	3526.40	3217.33	0.296	**0.019**	358.52	356.17	342.21	0.573	**<0.001**
40	3606.71	3892.30	3311.66	**0.037**	**0.035**	374.15	365.68	347.29	0.324	**0.0038**

Supplementary Table S13B. Back-transformed and pairwise comparison of weekly fetal growth biometrics across sum of very-long-even-chain saturated fatty acids tertiles in the NICHD Fetal Growth Studies-Singletons

GW	HC, mm					BPD, mm				
	Back-transformed geometric mean mm			Wald test for pairwise comparison p value		Back-transformed geometric mean mm			Wald test for pairwise comparison p value	
	1st tertile	2nd tertile	3rd tertile	2nd vs 1st tertile	3rd vs 1st tertile	1st tertile	2nd tertile	3rd tertile	2nd vs 1st tertile	3rd vs 1st tertile
10	51.89	51.94	48.6	0.976	0.071	13.45	12.75	11.8	0.180	**<0.001**
11	62.43	61.78	60.51	0.585	0.110	16.45	15.87	15.47	0.092	**0.004**
12	73.79	72.48	73.22	0.059	0.418	19.68	19.22	19.40	**0.025**	0.172
13	85.82	83.94	86.35	**0.005**	0.416	23.08	22.72	23.40	0.064	0.102
14	98.32	96.04	99.58	**0.011**	0.148	26.60	26.26	27.32	0.213	**0.005**
15	111.12	108.61	112.66	**0.016**	0.139	30.16	29.79	31.03	0.249	**0.005**
16	124.07	121.48	125.42	**0.018**	0.220	33.72	33.26	34.49	0.165	**0.019**
17	137.03	134.48	137.83	**0.023**	0.479	37.24	36.64	37.72	0.072	0.161
18	149.91	147.44	150.02	**0.041**	0.932	40.71	39.93	40.82	**0.032**	0.777
19	162.67	160.19	162.15	0.066	0.701	44.14	43.17	43.92	**0.017**	0.595
20	175.22	172.62	174.18	0.073	0.481	47.50	46.35	47.03	**0.008**	0.293
21	187.46	184.62	186.05	0.060	0.356	50.77	49.44	50.14	**0.003**	0.170
22	199.33	196.13	197.68	**0.039**	0.289	53.93	52.44	53.22	**<0.001**	0.127
23	210.77	207.13	209.00	**0.023**	0.271	56.98	55.34	56.25	**<0.001**	0.128
24	221.77	217.61	219.98	**0.014**	0.293	59.92	58.15	59.21	**<0.001**	0.166
25	232.34	227.62	230.60	**0.010**	0.346	62.75	60.88	62.10	**<0.001**	0.236
26	242.50	237.23	240.85	**0.008**	0.407	65.49	63.54	64.88	**<0.001**	0.309
27	252.33	246.54	250.73	**0.006**	0.448	68.16	66.16	67.56	**0.002**	0.338
28	261.89	255.68	260.26	**0.004**	0.446	70.80	68.78	70.11	**0.002**	0.284
29	271.27	264.77	269.49	**0.002**	0.395	73.41	71.41	72.53	**0.001**	0.161
30	280.36	273.72	278.36	**0.001**	0.338	75.99	74.03	74.83	**0.002**	0.068
31	289.02	282.38	286.78	**0.003**	0.315	78.47	76.60	77.03	**0.006**	**0.034**
32	297.10	290.61	294.65	**0.008**	0.320	80.81	79.05	79.16	**0.019**	**0.027**
33	304.43	298.23	301.89	**0.018**	0.334	82.96	81.34	81.25	**0.045**	**0.033**
34	310.84	305.06	308.39	**0.029**	0.353	84.85	83.39	83.33	0.073	0.060
35	316.24	310.96	314.04	**0.040**	0.407	86.45	85.17	85.41	0.107	0.205
36	320.83	315.98	318.56	0.082	0.452	87.80	86.69	87.30	0.197	0.597
37	324.90	320.34	321.62	0.147	0.337	88.94	88.02	88.71	0.340	0.826
38	328.76	324.27	322.90	0.168	0.083	89.96	89.27	89.35	0.488	0.553
39	332.74	328.04	322.09	0.196	**0.003**	90.92	90.51	88.91	0.717	0.070
40	337.20	331.89	318.91	0.415	**0.007**	91.89	91.86	87.15	0.990	**0.020**

Supplementary Table S14. Association between individual SFA and fetal growth throughout between 10-40 weeks gestation using cubic spline model with gestational weeks after adjusting for maternal family history of diabetes

SFAs in tertiles	Estimated Fetal Weight, g	Abdominal circumference, mm	Femur length, mm	Head circumference, mm	Biparietal diameter, mm	HC/AC ratio
	Globa test (after adjusting for maternal ge, ethnicity/race, nulliparity, pre-prengnacy body mass index and fetal sex) with interaction term SFA*GA					
	Likelihood ratio with Bonferroni correction	Likelihood ratio with Bonferroni correction	Likelihood ratio with Bonferroni correction	Likelihood ratio with Bonferroni correction	Likelihood ratio with Bonferroni correction	Likelihood ratio with Bonferroni correction
Myristic acid (14:0)	**<0.00001**	**<0.00001**	**0.011**	**<0.0001**	**<0.00001**	**<0.00001**
Pentadecanoic acid (15:0)	**<0.00001**	**<0.00001**	**0.005**	**<0.00001**	**<0.00001**	**<0.00001**
Palmitic acid (16:0)	0.171	**0.021**	1.00	1.00	1.00	<0.00001
Heptadecanoic acid (17:0)	**<0.00001**	**<0.001**	**<0.00001**	**<0.00001**	**<0.00001**	1.00
Stearic acid (18:0)	**<0.0001**	**<0.00001**	**<0.00001**	**<0.00001**	**<0.00001**	**<0.00001**
Arachidic acid (20:0)	0.129	1.00	**0.002**	**0.005**	**<0.00001**	**0.019**
Behenic acid (22:0)	**<0.00001**	**<0.00001**	0.226	**<0.00001**	**<0.00001**	**<0.00001**
Lignoceric acid (24:0)	**<0.00001**	**<0.00001**	**0.031**	**<0.00001**	**<0.00001**	**<0.00001**
Sum of even-chain SFAs (14:0 + 16:0 + 18:0)	1.00	**<0.00001**	**<0.00001**	**<0.00001**	**<0.00001**	**0.0001**
Sum of odd-chain SFAs (15:0 + 17:0)	**<0.00001**	1.00	**0.0039**	**<0.00001**	**<0.00001**	0.178
Sum of very-long-even-chain SFAs (20:0 + 22:0 + 24:0)	**<0.00001**	**<0.00001**	0.073	**<0.00001**	**<0.00001**	**<0.00001**

Abbreviations: GA, gestational age, SFA, saturated fatty acids; HC/AC ratio, head circumference/abdominal circumference ratio.

Supplementary Table S15. Association between individual SFA and fetal growth throughout between 10-40 weeks gestation using cubic spline model with gestational weeks after adjusting for maternal random glucose level at visit 0

SFAs in tertiles	Estimated Fetal Weight, g	Abdominal circumference, mm	Femur length, mm	Head circumference, mm	Biparietal diameter, mm	HC/AC ratio
	Globa test (after adjusting for maternal ge, ethnicity/race, nulliparity, pre-prengnacy body mass index and fetal sex) with interaction term SFA*GA					
	Likelihood ratio with Bonferroni correction	Likelihood ratio with Bonferroni correction	Likelihood ratio with Bonferroni correction	Likelihood ratio with Bonferroni correction	Likelihood ratio with Bonferroni correction	Likelihood ratio with Bonferroni correction
Myristic acid (14:0)	**<0.00001**	**<0.00001**	**0.012**	**<0.00001**	**<0.00001**	**<0.00001**
Pentadecanoic acid (15:0)	**<0.00001**	**<0.00001**	**0.005**	**<0.00001**	**<0.00001**	**<0.00001**
Palmitic acid (16:0)	0.206	**0.041**	1.00	1.00	1.00	**<0.00001**
Heptadecanoic acid (17:0)	**<0.00001**	**0.001**	**<0.00001**	**<0.00001**	**<0.00001**	1.00
Stearic acid (18:0)	**<0.00001**	**<0.00001**	**<0.00001**	**<0.00001**	**<0.00001**	**<0.00001**
Arachidic acid (20:0)	0.140	0.521	**0.002**	**<0.001**	**<0.00001**	**0.026**
Behenic acid (22:0)	**<0.00001**	**<0.00001**	0.2160	**<0.00001**	**<0.00001**	**<0.00001**
Lignoceric acid (24:0)	**<0.00001**	**<0.00001**	**0.030**	**<0.00001**	**<0.00001**	**<0.00001**
Sum of even-chain SFAs (14:0 + 16:0 + 18:0)	**<0.00001**	**<0.00001**	**<0.00001**	**<0.00001**	**<0.00001**	**<0.00001**
Sum of odd-chain SFAs (15:0 + 17:0)	**<0.00001**	1.00	**0.004**	**<0.00001**	**<0.00001**	0.119
Sum of very-long-even-chain SFAs (20:0 + 22:0 + 24:0)	**<0.00001**	**<0.00001**	0.061	**<0.00001**	**<0.00001**	**<0.00001**

Abbreviations: GA, gestational age, SFA, saturated fatty acids; HC/AC ratio, head circumference/abdominal circumference ratio.

Supplementary Table S16. Association between individual SFA and fetal growth throughout between 10-40 weeks gestation using cubic spline model with gestational weeks after adjusting for maternal total cholesterol level at visit 0

SFAs in tertiles	Estimated Fetal Weight, g	Abdominal circumference, mm	Femur length, mm	Head circumference, mm	Biparietal diameter, mm	HC/AC ratio
	Globa test (after adjusting for maternal ge, ethnicity/race, nulliparity, pre-prengnacy body mass index and fetal sex) with interaction term SFA*GA					
	Likelihood ratio with Bonferroni correction	Likelihood ratio with Bonferroni correction	Likelihood ratio with Bonferroni correction	Likelihood ratio with Bonferroni correction	Likelihood ratio with Bonferroni correction	Likelihood ratio with Bonferroni correction
Myristic acid (14:0)	**<0.00001**	**<0.00001**	**0.013**	**<0.00001**	**<0.00001**	**<0.00001**
Pentadecanoic acid (15:0)	**<0.00001**	**<0.00001**	**0.005**	**<0.00001**	**<0.00001**	**<0.00001**
Palmitic acid (16:0)	0.202	**0.016**	1.00	1.00	1.00	**<0.00001**
Heptadecanoic acid (17:0)	**<0.00001**	**0.003**	**<0.00001**	**<0.00001**	**<0.00001**	0.587
Stearic acid (18:0)	**<0.00001**	**<0.00001**	**<0.00001**	<0.00001	<0.00001	<0.00001
Arachidic acid (20:0)	0.131	0.600	**0.002**	<0.001	<0.00001	0.003
Behenic acid (22:0)	**<0.00001**	**<0.00001**	0.230	<0.00001	<0.00001	<0.00001
Lignoceric acid (24:0)	**<0.00001**	**<0.00001**	**0.027**	**<0.00001**	**<0.00001**	**<0.00001**
Sum of even-chain SFAs (14:0 + 16:0 + 18:0)	**<0.00001**	**<0.00001**	**<0.00001**	**<0.00001**	**<0.00001**	**<0.00001**
Sum of odd-chain SFAs (15:0 + 17:0)	**<0.00001**	1.00	**0.004**	**<0.00001**	**<0.00001**	**0.009**
Sum of very-long-even-chain SFAs (20:0 + 22:0 + 24:0)	**<0.00001**	**<0.00001**	0.066	**<0.00001**	**<0.00001**	**<0.00001**

Abbreviations: GA, gestational age, SFA, saturated fatty acids; HC/AC ratio, head circumference/abdominal circumference ratio.

Supplementary Table S17. Association between individual SFA and fetal growth throughout between 10-40 weeks gestation using cubic spline model with gestational weeks after adjusting for subgroup SFAs at visit 0

SFAs in tertiles	Estimated Fetal Weight, g	Abdominal circumference, mm	Femur length, mm	Head circumference, mm	Biparietal diameter, mm	HC/AC ratio
	Globa test (after adjusting for maternal ge, ethnicity/race, nulliparity, pre-prengnacy body mass index and fetal sex) with interaction term SFA*GA					
	Likelihood ratio with Bonferroni correction	Likelihood ratio with Bonferroni correction	Likelihood ratio with Bonferroni correction	Likelihood ratio with Bonferroni correction	Likelihood ratio with Bonferroni correction	Likelihood ratio with Bonferroni correction
Myristic acid (14:0)	0.99	**0.005**	**0.007**	**<0.00001**	**<0.00001**	**<0.00001**
Pentadecanoic acid (15:0)	**<0.00001**	**<0.00001**	**0.003**	**<0.00001**	**<0.00001**	**<0.00001**
Palmitic acid (16:0)	0.258	**0.005**	0.99	0.99	0.99	**<0.00001**
Heptadecanoic acid (17:0)	**<0.00001**	**<0.00001**	**<0.00001**	**<0.00001**	**<0.00001**	0.550
Stearic acid (18:0)	**<0.00001**	**<0.00001**	**<0.00001**	**<0.00001**	**<0.00001**	**<0.00001**
Arachidic acid (20:0)	0.99	0.99	**0.0004**	**0.037**	**<0.00001**	**0.020**
Behenic acid (22:0)	0.124	**<0.00001**	0.058	**<0.00001**	**<0.00001**	**<0.00001**
Lignoceric acid (24:0)	**<0.00001**	**<0.00001**	**0.022**	**<0.00001**	**<0.00001**	**<0.00001**
Sum of even-chain SFAs (14:0 + 16:0 + 18:0)	0.99	0.99	**<0.00001**	**<0.00001**	**0.0006**	**<0.00001**
Sum of odd-chain SFAs (15:0 + 17:0)	**<0.00001**	**<0.00001**	**0.001**	**<0.00001**	**<0.00001**	0.071
Sum of very-long-even-chain SFAs (20:0 + 22:0 + 24:0)	**<0.00001**	**<0.00001**	**0.007**	**<0.00001**	**<0.00001**	**<0.00001**

Abbreviations: GA, gestational age, SFA, saturated fatty acids; HC/AC ratio, head circumference/abdominal circumference ratio.
